# Comparative Performance of High-Yielding European Wheat Cultivars Under Contrasting Mediterranean Conditions

**DOI:** 10.3389/fpls.2021.687622

**Published:** 2021-06-29

**Authors:** Valter Jário de Lima, Adrian Gracia-Romero, Fatima Zahra Rezzouk, Maria Carmen Diez-Fraile, Ismael Araus-Gonzalez, Samuel Henrique Kamphorst, Antonio Teixeira do Amaral Júnior, Shawn C. Kefauver, Nieves Aparicio, Jose Luis Araus

**Affiliations:** ^1^Laboratório de Melhoramento Genético Vegetal, Centro de Ciências e Tecnologias Agropecuárias (CCTA), Universidade Estadual do Norte Fluminense Darcy Ribeiro – UENF, Campos dos Goytacazes, Brazil; ^2^Integrative Crop Ecophysiology Group, Plant Physiology Section, Faculty of Biology, University of Barcelona, Barcelona, Spain; ^3^AGROTECNIO (Center for Research in Agrotechnology), Lleida, Spain; ^4^Agro-Technological Institute of Castilla and Leon (ITACyL), Valladolid, Spain

**Keywords:** ideotype, drought, nitrogen fertilization, phenology, stay-green

## Abstract

Understanding the interaction between genotype performance and the target environment is the key to improving genetic gain, particularly in the context of climate change. Wheat production is seriously compromised in agricultural regions affected by water and heat stress, such as the Mediterranean basin. Moreover, wheat production may be also limited by the nitrogen availability in the soil. We have sought to dissect the agronomic and physiological traits related to the performance of 12 high-yield European bread wheat varieties under Mediterranean rainfed conditions and different levels of N fertilization during two contrasting crop seasons. Grain yield was more than two times higher in the first season than the second season and was associated with much greater rainfall and lower temperatures. However, the nitrogen effect was rather minor. Genotypic effects existed for the two seasons. While several of the varieties from central/northern Europe yielded more than those from southern Europe during the optimal season, the opposite trend occurred in the dry season. The varieties from central/northern Europe were associated with delayed phenology and a longer crop cycle, while the varieties from southern Europe were characterized by a shorter crop cycle but comparatively higher duration of the reproductive period, associated with an earlier beginning of stem elongation and a greater number of ears per area. However, some of the cultivars from northern Europe maintained a relatively high yield capacity in both seasons. Thus, KWS Siskin from the UK exhibited intermediate phenology, resulting in a relatively long reproductive period, together with a high green area throughout the crop cycle.

## Introduction

There is an increasing demand for food production as a result of world population growth (Prasad et al., 2008; Holman et al., 2016; Kefauver et al., 2017). Wheat is one of the most important crops for food security, accounting for about 20% of the total diet worldwide (Shiferaw et al., 2013). Large quantities of wheat are produced in Europe, supplying around 35% of global demand; however, the yield variation between European regions can amount to 6 Mg ha^−1^ (Senapati and Semenov, 2020). The main causes of differences in crop yield are due to frequent heat and drought stresses in southern Europe and low temperatures in northern Europe, together with soil nutritional deficiencies (Wang and Frei, 2011; Senapati and Semenov, 2020), which are mainly related to nitrogen availability (Savin et al., 2015). Moreover, it is increasingly common for agricultural regions, such as the European Mediterranean, to be affected by water and/or thermal stress due to climate change (Soriano et al., 2018; Senapati and Semenov, 2020).

Abiotic stresses, such as water stress, heat, and nutritional deficiency, can negatively affect crop yields and, eventually, world food security (Wang and Frei, 2011; Aprile et al., 2013; Holman et al., 2016; Sehgal et al., 2018). In the European Mediterranean basin, the production of wheat is mostly limited by water stress due to low precipitation and elevated levels of evapotranspiration caused by high temperatures (Oweis et al., 2000; Tambussi et al., 2007; Lobell et al., 2008). Indeed, the combination of water deficit and heat is known as drought, which is considered the main stress factor limiting productivity in Mediterranean agrosystems (Soriano et al., 2018). Nitrogen deficiency also plays a significant yield-limiting role, particularly when combined with drought (Abeledo et al., 2008; Gastal et al., 2015; Cossani and Sadras, 2018). In fact, nitrogen has been considered the most important nutrient for increasing cereal production in recent decades (Good and Beatty, 2011). Modern agriculture is characterized by the increased consumption and cost of nitrogen fertilizers needed to maintain crop productivity (Brennan et al., 2014; Gastal et al., 2015). Nevertheless, it has been highlighted that excessive and continuous nitrogen fertilization triggers eutrophication conditions, causing negative environmental consequences (Good and Beatty, 2011; Gastal et al., 2015). In addition, excessive nitrogen fertilization under drought conditions may cause a penalty in yield, a phenomenon that is known as haying-off (van Herwaarden et al., 1998), which for wheat is predicted to increase in the Mediterranean under future climate conditions (Nuttall et al., 2012). Therefore, it is important to optimize the use of nitrogen through the adoption of strategies that benefit farmers economically and reduce environmental impacts, directing farmers toward the adoption of more sustainable agricultural practices (Gastal et al., 2015; Kefauver et al., 2017; Vicente et al., 2019).

After the Green Revolution, increases in wheat production have been mainly driven by the improvement of management techniques, including nitrogen fertilization, and breeding of new cultivars (Wang and Frei, 2011; Holman et al., 2016). These breeding programs introduced semi-dwarf wheat varieties that possess higher yield potential due to an increased harvest index and better lodging tolerance (Reif et al., 2005). In the case of Europe, despite evidence of genetic advances in recent decades, there is still an insufficient understanding of the interaction of genotype, target environment, and management conditions (GxExM) with the physiological characteristics behind genotypic adaptation (Senapati and Semenov, 2020). This is a very relevant issue, considering that climate change is a reality that is rapidly modifying environmental conditions throughout Europe (Olesen et al., 2012; Blanco et al., 2017; Agovino et al., 2019), shifting toward scenarios that somewhat resemble Mediterranean environments in terms of increased temperatures, the occurrence of drought events, and interannual variability in environmental conditions (Olesen et al., 2012; Neset et al., 2019). On the other hand, genetic advances in Mediterranean countries have been less evident than in the temperate regions of Europe (Soriano et al., 2018; Rufo et al., 2019), in spite of the fact that germplasm originating in Europe has been one of the main sources of new cultivar development (Sanchez-Garcia et al., 2013; Chairi et al., 2018, 2020; Rufo et al., 2019). As the performance of newly developed cultivars relies on the magnitude of GxExM interactions (Araus and Kefauver, 2018), the use of resilient germplasm, together with adequate phenotyping approaches, is the only way to find solutions and improve the efficiency of breeding in the face of climate change (Araus and Kefauver, 2018).

To date, phenological adaptation is one of the main features when considering germplasm performance to Mediterranean conditions, where crop escape (i.e., where the impact of abiotic stresses is avoided during the last stages of the crop) has been a successful strategy for many years (Soriano et al., 2018; Rufo et al., 2019; Sallam et al., 2019). In addition, early sowing has been a good strategy under Mediterranean conditions (Hunt et al., 2019), providing that the pattern of rainfall (not too much nor too little) allows it. However, not only shorter crop duration and earlier maturity may provide better genotypic adaptation to Mediterranean conditions, but differences in the relative duration of vegetative and reproductive stages, as well as in growth patterns, should be considered. In that sense, the use of remote-sensing sensors has become an important foundation for both precision crop monitoring and phenotyping due to their wide range of applications and their nondestructive nature (Araus and Cairns, 2014). In this regard, the formulation of vegetation indices, measured through multispectral sensors and, more recently, with conventional digital cameras, has proven to be a very useful option for rapid and nondestructive screening of the responses of cereal to a water regimen, heat (Araus and Kefauver, 2018; Gracia-Romero et al., 2019), and N fertilization (Cabrera-Bosquet et al., 2011; Kefauver et al., 2017; Fernandez-Gallego et al., 2019). The normalized difference vegetation index (NDVI) is one of the most well-established multispectral vegetation indices (Marti et al., 2007; Cabrera-Bosquet et al., 2011; Duan et al., 2017), while, among the estimates derived from RGB images, the greener area (GGA) has proven its performance in assessing crop growth, photosynthetic area, and canopy senescence (Yousfi et al., 2016, 2019; Fernandez-Gallego et al., 2019).

This study compares the performance of a set of modern wheat cultivars that rank among the highest yielders in different European countries under the Mediterranean conditions of northwest Spain. We sought to dissect the agronomic and physiological traits related to the performance of high-yield European bread wheat varieties grown for two consecutive seasons under rainfed conditions and different levels of N availability. The main objective was to identify the ideotype characteristics in wheat that confer resilience to fluctuating Mediterranean conditions. The second objective consisted of assessing the adequacy of current nitrogen fertilization conditions and their interaction with climate. This research is associated with the European Consortium for Open Field Experimentation (ECOFE), which covers all major climatological regions of Europe. ECOFE aims to develop systematic investigations of the interactions between a plant genotype, environment, and agricultural management, i.e., studies of high-yielding wheat varieties across a range of farming practices and locations under highly standardized conditions.^1^ In that sense, the present study was performed in one of the experimental stations of the project.

## Materials and Methods

### Plant Material, Site Description, and Growing Conditions

Twelve commercial European varieties of bread wheat (*Triticum aestivum* L.) of different European origins (Table 1) were selected for their high yield traits in their country of provenance. The experiment was carried out during two consecutive crop growing seasons (2017–2018 and 2018–2019) at the Zamadueñas Station, Valladolid, (41° 41′N, 04° 42′W, 700-m altitude), which belongs to the Instituto Tecnológico Agrario de Castilla y León (ITACyL).

**Table 1:** Bread wheat varieties used in the experiment, with their supplying institution and country of origin.

Variety	Origin		
	Country	Provider Institution	Justification for selection
Bologna	Italy	Department of Agricultural Sciences, University of Bologna	High yield and hard kernel
Chambo	Spain	Institute of Agrifood Research and Technology – IRTA	High yield and rust resistance
Soberbio	Spain	Instituto Tecnológico Agrario de Castilla y León	High yield and grain quality characteristics
Henrik	Belgium	Ghent University - Flanders Research Institute for Agriculture, Fisheries and Food (ILVO)	High and stable yield over years, and largest growing area
Benchmark	Germany	Hannover University	Among the highest yielding varieties in Lower Saxony
RGT Reform	Germany	Hohenheim University	Widely adopted by farmers, high yield, and grain quality characteristics
JB Diego	Ireland	National University of Ireland, Galway	High yield under cultivation conditions in western Ireland, and high grain weight
KWS Lili^∗^	Ireland	Teagasc - Agriculture and Food Development Authority	High yielding and stable variety under Irish conditions
Hondia	Poland	Institute of Soil Science and Plant Cultivation-State Research Institute	New variety, high yield, and grain quality characteristics
Julius	Sweden	Swedish University of Agricultural Sciences	High yielding and the first milling variety
CH-Nara	Switzerland	Agroscope	Widely adopted by farmers, high yield, baking quality, and other grain quality characteristics
KWS Siskin	United Kingdom	Rothamsted Research	New high yielding and first milling variety, with early stem elongation and canopy closure

∗ In 2018/19, the KWS-Lili variety was replaced by the Bennington variety.

**Figure 1:**
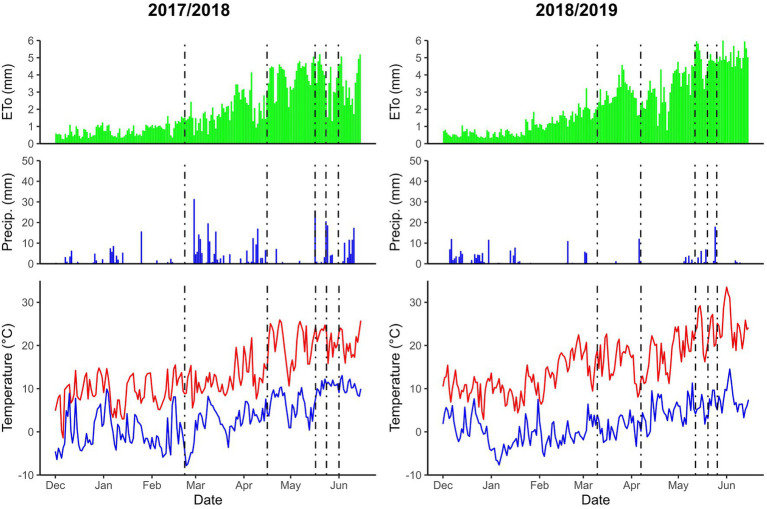
Accumulated monthly precipitation, maximum and minimum temperature, and potential evapotranspiration (ETo) in Zamadueñas for the 2017/18 and 2018/19 growing seasons. The dashed lines in the graphs correspond to the stages of tillering, stem elongation, booting, heading, and anthesis, respectively.

The monthly averages of temperature, precipitation, and potential evapotranspiration are shown in Figure 1. Climatic data were recorded from a meteorological station located in the experimentation field of the Spanish platform, SIAR (Servicio de Información Agroclimática para el Regadío, www.siar.es).

The genotypes were grown under rainfed conditions and three levels of top-dressing nitrogen fertilization: N100 (optimum nitrogen fertilization recommended in the zone), N50 (50% of the optimum N dose recommended in the zone), and N130: (30% above the optimum). Soil in the first season trial was silt-loam, with 1.10% organic matter and a pH of 8.5, while in the second season, it was loam, with 2.98% organic matter and, again, a pH of 8.5 (Supplementary Table 1). The soils of Castilla y León, where 50% of Spanish wheat is produced, are characterized by basic pH. The sowing was carried out during fall as soon as the climatic conditions allowed. In the 2017/18 crop season, the previous September and most of October were very dry, but 12-mm rainfall occurred at the end of October, which allowed for adequate soil preparation and planting on November 2, 2017. In the case of the 2018/19 crop season, sowing was delayed till November 29, 2018, because the dry September and October, followed by frequent rains during the first half of November, prevented planting.

For the two growing seasons evaluated, the experimental design was a split plot into RCBD (a randomized complete block design), with nitrogen levels allocated to main plots and genotypes to subplots, with three replicates consisting of 12-m long and 1.5-m wide plots, with seven rows and 21 cm between them. Therefore, a total of 108 plots, of 18 m^2^ each, were established. The sowing rate was 450 seed m^−2^. Nitrogen fertilizer was applied as follows; before sowing, the field was fertilized with 300 kg ha^−1^ of NPK (8–15–15), and two further top-dressing fertilizer applications were made during wheat cultivation. The first top fertilizer application was carried out during tillering, approximately 3 months after sowing, with 150 kg ha^−1^ of calcium ammonium nitrate (27% N) in all treatments. In the second application, 4 months after sowing, N50 was not fertilized, and 150 kg ha^−1^ and 240 kg ha^−1^ of ammonium nitrosulfate were applied to N100 and N130, respectively. Appropriate herbicides, pesticides, and insecticides were applied according to normal practices prevalent in the region. Harvesting took place on July 31, 2018, and July 15, 2019, respectively.

### Agronomic Traits

For each plot, yield components were measured at crop maturity by sampling plants in two 50-cm central-row sections. Then, the number of spikes per square meter (SM) was calculated. The aerial portion of the plants was weighed after being oven-dried at 70°C for 48 h, and the total dry weight of the aerial biomass (BDW) was determined. Later, a subsample of around 20% was taken, and, from it, thousand grain weight (TGW) and grains per spike (GS) were determined. The plots were harvested mechanically, and grain yield (GY) was determined for each plot and adjusted to a 10% moisture level.

### Phenological Stage

Phenological stages were evaluated according to the Zadoks phenological scale (Zadoks et al., 1974). The stages of tillering, stem elongation, booting, heading, anthesis, and middle grain filling were visually determined and expressed in days after sowing (DAS).

### Vegetation Indices

The normalized difference vegetation index (NDVI) was determined in each plot using a portable spectroradiometer (GreenSeeker, Trimble, United States). The Greenseeker is an active sensor, a spectroradiometer that uses NIR- and RED-modulated illumination and detection and is not affected by ambient radiation. Because active sensors such as GreenSeeker have their own source of light and are not affected by ambient radiation, they can be used at any time of day or night and in different areas and different ambient radiation conditions. The wavelength of Greenseeker’s bands was in the visible near IR (770 nm) and red (660 nm) regions of the spectrum. The full-width half-maximum bandwidth was approximately 25 nm. The reflectance in the field of the wheat crop crown was obtained by passing the sensor over the middle of each plot at a constant height of approximately 50 cm above across the canopy, with a field of view of about 25 cm. NDVI is formulated using the following equation:

$$\mathrm{NDVI}=\left(\mathrm{NIR}-R\right)/\left(\mathrm{NIR}+R\right)$$ (1)

where R is the reflectance in the red range and NIR is the reflectance in the near-infrared range.

**Figure 2:**
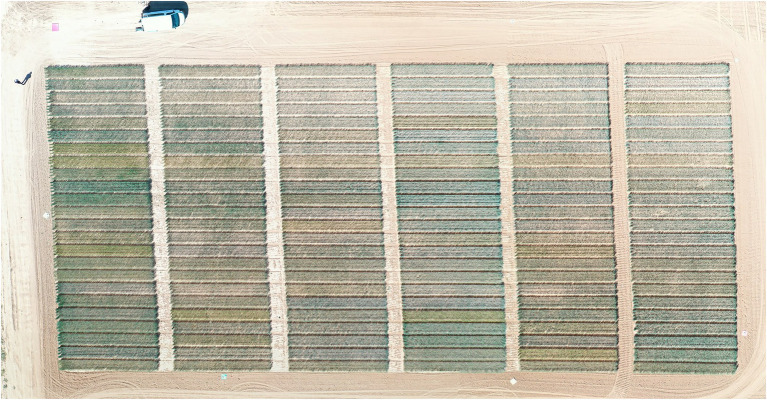
Orthomosaic of aerial images of the 2018/2019 trial corresponding to the anthesis stage (GGA9).

A conventional digital RGB image was taken per plot, placing the camera at approximately 100 cm above the canopy, in a zenith plane, and focusing close to the center of each plot. The images were acquired with a Sony ILCE-QX1 (Sony Europe Limited, Brooklands, United Kingdom), a digital camera of 20.1-megapixel resolution, equipped with a 23.2 mm × 15.4 mm sensor size (type CMOS Exmor HD) and using a 16-mm focal lens and an exposure time of 1/60 s. Images of 4,608 × 3,456 pixels were captured and stored in the JPG format using the RGB color standard. In order to capture the entire plot, the last two series of RGB images collected during 2018/19 were captured as aerial images by using an eight-rotor Mikrokopter Oktokopter XL 6S (HiSystems GmbH, Moomerland, Germany), equipped with a 16-megapixel Lumix GX7 (Panasonic, Osaka, Japan), a digital mirrorless RGB camera of 16.0 megapixels with an image sensor size of 17.3 × 13.0 mm (type Live MOS) using a 20-mm focal lens with an exposure time of 1/8,000 s. The ground sample distance of the aerial images for a flight at a 50-m altitude was a 0.941 cm pixel^−1^, and the area captured in each image corresponded to 1405 m^2^. To correct the effect of pitch and roll movements of the drone during the flight, an active two-servo gimbal was used to steady the camera. Preprocessed aerial images with at least 80% overlap were combined to obtain an accurate orthomosaic (Figure 2) by producing a 3D reconstruction with Agisoft PhotoScan Professional software (Agisoft LLC, St. Petersburg, Russia, www.agisoft.com). Then, regions of interest corresponding to each plot were segmented and exported using the MosaicTool (Shawn C. Kefauver, https://integrativecropecophysiology.com/software-development/mosaictool/, https://gitlab.com/sckefauver/MosaicTool, University of Barcelona, Barcelona, Spain), integrated as a plug-in for the open source image analysis platform FIJI (Fiji is Just ImageJ; http://fiji.sc/Fiji). The processing of the RGB images for the calculation of the vegetation indices in relation to different color properties of potential interest was performed with the MosaicTool that includes a JAVA8 version of Breedpix 2.0 (Jaume Casadesús, https://bio-protocol.org/e1488, IRTA, Lleida, Spain). The measured vegetation index was the greener green area (GGA). GGA is the percentage of pixels in the image with a range of hues from 80 to 180°, including yellowish-green color. NDVI measurements and RGB images were collected on fully sunny days, between 10:00 a.m. and 3:00 p.m. solar time, at different phenological stages as shown in Table 2.

**Table 2:** Dates of measurements of normalized difference vegetation index (NDVI) and greener area (GGA) indices, presented as the calendar date, days after sowing (DAS), developmental scale (Zadoks et al., 1974), and corresponding phenological stage.

Season	Date	DAS	Vegetation indices		Zadoks scale	Phenological stage
2017/18	22-February	112		GGA1	20–29	Tillering
	26-March	144		GGA2		
	6-April	153	NDVI1		30	Stem elongation
	17-April	166	NDVI2	GGA3		
	17-May	196	NDVI3	GGA4	45	Booting
	31-May	210		GGA5	65	Anthesis
	6-June	216	NDVI4			
	20-June	230	NDVI5	GGA6	75	Middle grain filling
2018/19	18-February	74		GGA1	13	3 leaves unfolding
	5-March	96		GGA2	20–29	Tillering
	21-March	12	NDVI1	GGA3		
	3-April	124	NDVI2	GGA4	30	Stem elongation
	26-April	148	NDVI3	GGA5		
	6-May	158	NDVI4	GGA6	45	Booting
	14-May	166	NDVI5	GGA7	55	Heading
	24-May	176		GGA8	65	Anthesis
	28-May	180	NDVI6			
	29-May	181		GGA9^∗^		
	11-June	194		GGA10^∗^	75	Middle grain filling

*Unlike the other GGA indices, which were calculated from a single image, these indices were calculated from segmented images. The methodological details of image acquisition and processing are found in the “Vegetation Indices” section of MATERIALS AND METHODS.

### Statistical Analysis

Statistical analyses were performed using the open-source software R (R Core Team, 2017) and RStudio 4.0.2 (R Core Team, 2018). The data were subjected to split-plot analysis of variance, with the nitrogen level allocated to main plots and genotypes to sub-plots, with the aim of testing the effects of N availability, genotypes, and their interactions for each growing season. In addition, a joint analysis of the growing seasons was also carried out, considering the effect of the year and its interactions with N and a genotype. The comparisons of means between levels of N and between genotypes were performed according to the Tukey’s honestly significant difference (HSD) test when the corresponding effect of ANOVA was significant according to the F test (*p* < 0.05). To analyze the relationship between the evaluated traits and the GY, Pearson’s bivariate correlation coefficients were calculated. In addition, a principal component analysis (PCA) was carried out for each growing season. For a further dissection of the genotypic effect on these correlations, the genetic correlations (*r_g_*) were calculated using Meta-R (Multi-Environment Trial Analysis with R for Windows), version 6.01 (Alvarado et al., 2017). The genetic correlation was calculated as follows:

$$r_{g=\frac{COV_{GY*X}}{\sqrt{\sigma_{GY}^{2}+\sigma_{X}^{2}}}}$$ (2)

where COV_GY*X_ is the covariance between GY and trait x, $\sigma_{GY}^{2}$ is the variance component of GY, and $\sigma_{X}^{2}$ is the variance component of trait x.

## Results

The effect of the growing season (year) was significant for both GY and all yield components (Supplementary Table 2). Grain yield (GY) showed a strong decrease during the 2018/19 crop growing season (averaged value across genotypes and nitrogen conditions of 2.52 Mg ha^−1^) relative to the 2017/18 season (7.95 Mg ha^−1^). The general effect of nitrogen fertilization was significant only for GY (Supplementary Table 2). However, only during 2017/18, nitrogen fertilization significantly affected GY, with N50 exhibiting lower values compared with N100 and N130 (Table 3).

**Table 3:** Summary of the analysis of variance (ANOVA) for nitrogen fertilization (N) and genotypic (G) effects (upper part of the table), mean values for GY and yield components within each nitrogen fertilizer level (middle part), and mean genotypic values for GY (lower part).

Traits	2017/18			2018/19		
	Level of significance (ANOVA)					
	N	G	N × G	N	G	N × G
GY	0.008^∗∗^	0.000^∗∗∗^	0.123^ns^	0.679^ns^	0.000^∗∗∗^	0.933^ns^
TGW	0.551^ns^	0.000^∗∗∗^	0.221^ns^	0.198^ns^	0.000^∗∗∗^	0.284^ns^
BDW	0.941^ns^	0.056^ns^	0.078^ns^	0.770^ns^	0.735^ns^	0.135^ns^
SM	0.897^ns^	0.013^∗^	0.113^ns^	0.752^ns^	0.000^∗∗∗^	0.892^ns^
GS	0.048^∗^	0.000^∗∗∗^	0.879^ns^	0.827^ns^	0.006^∗∗^	0.170^ns^
	**Significance of the mean ± standard deviation of nitrogen fertilizer levels**					
	**N50**	**N100**	**N130**	**N50**	**N100**	**N130**
GY	7.41 ± 0.87b	8.35 ± 0.89a	8.09 ± 0.98a	2.39 ± 0.66a	2.46 ± 0.8a	2.72 ± 0.73a
TGW	35.21 ± 3.27a	34.78 ± 2.97a	34.89 ± 3.2a	26.97 ± 4.39a	28.26 ± 4.51a	29.60 ± 4.08a
BDW	1.25 ± 0.28a	1.35 ± 0.29a	1.34 ± 0.3a	0.56 ± 0.13a	0.57 ± 0.14a	0.59 ± 0.15a
SM	458.58 ± 69.99a	464.47 ± 92.44a	453.33 ± 74.34a	291.31 ± 79.78a	301.36 ± 90.64a	321.81 ± 78.19a
GS	45.89 ± 7.67a	43.22 ± 6.48a	45.28 ± 6.53a	21.87 ± 6.28a	23.16 ± 5.64a	22.31 ± 6.84a
**Genotype**	**Significance of genotypes for GY (HSD; mean ± standard deviation)**					
	**N50**	**N100**	**N130**	**Genotype**	**General**
Bologna	6.56 ± 0.41cd	6.82 ± 0.36c	6.44 ± 0.51d	Bologna	2.79 ± 1.02ab
Chambo	7.13 ± 0.88abcd	8.44 ± 0.20ab	9.15 ± 0.50ab	Chambo	2.89 ± 0.51ab
Soberbio	7.71 ± 0.80abc	8.33 ± 0.65ab	8.30 ± 0.41abc	Soberbio	3.15 ± 0.65a
Henrik	7.73 ± 0.29abc	8.66 ± 0.33ab	8.79 ± 0.24ab	Henrik	2.92 ± 0.72ab
Benchmark	8.09 ± 0.16ab	8.54 ± 0.50ab	8.74 ± 0.45ab	Benchmark	2.00 ± 0.44b
RGT Reform	8.12 ± 0.61ab	9.17 ± 0.39ab	8.76 ± 0.02ab	RGT Reform	2.28 ± 0.62ab
JB Diego	8.31 ± 0.33ab	9.25 ± 0.20ab	8.37 ± 0.63ab	JB Diego	2.17 ± 0.66ab
KWS Lilli	7.03 ± 0.33bcd	8.41 ± 0.03ab	8.01 ± 0.26abc	Bennington	2.33 ± 0.41ab
Hondia	6.89 ± 0.46bcd	8.06 ± 0.43bc	7.46 ± 0.81bcd	Hondia	1.92 ± 0.59b
Julius	6.97 ± 0.39bcd	8.11 ± 0.46b	7.90 ± 0.54abcd	Julius	2.18 ± 0.65ab
CH-Nara	5.89 ± 0.21d	6.85 ± 0.39c	6.52 ± 0.18cd	CH-Nara	2.55 ± 0.57ab
KWS Siskin	8.48 ± 0.45a	9.55 ± 0.69a	9.23 ± 0.49a	KWS Siskin	2.94 ± 0.67ab

GY, grain yield (Mg ha^−1^); TGW, thousand grain weight; BDW, biomass dry weight; SM, spikes m^−2^; GS, grains spike^−^1. ^*^For the 2018/19 season, genotypic means of GY were only calculated for all three fertilizer levels combined because the nitrogen fertilizer treatments had significant effect. Means across nitrogen levels (in the row) and across genotypes (in the column), followed by different letters, are significantly different (*p* < 0.05) according to the Tukey’s honestly significant difference (HSD) test. Levels of significance for the ANOVA: ns, not significant; ^∗^*p* < 0.05; ^∗∗^*p* < 0.01; ^∗∗∗^*p* < 0.001.

The general effect of the genotype was significant for GY and all yield components, excluding biomass (BDW; Supplementary Table 2). Also, within each N fertilizer treatment, the genotypic effect in the 2017/18 season was significant for GY as well as for all the yield components evaluated, excluding biomass (BDW). A significant genotype-by-year interaction was observed for GY, TGW, and GS (Supplementary Table 2).

In the 2017/18 season, except for Henrik, Benchmark, and Chambo, which reached their greatest yields at N130, the highest GY was achieved under the recommended (N100) fertilization with the majority of cultivars (Table 3). The average reductions in GY under the N50 and N130 treatments relative to N100 were 0.9 and 0.2 Mg ha^−1^, respectively. KWS Lili, Chambo, Hondia, and Julius showed the greatest losses in GY due to reduced fertilization (N50), being 1.4, 1.3, 1.2, and 1.1 Mg ha^−1^, respectively. On the other hand, JB Diego had the most reduced yield (0.9 M ha^−1^) due to excess fertilizer, when comparing N100 to N130. During the same 2017/18 season, KWS Siskin produced the best GY under the three fertilizer levels. However, its GY values were only statistically different from CH-Nara, Bologna, and Hondia within each of the three fertilization levels, from Julius in the N50 and N100 treatments and from KWS Lili in the N50 treatment (Table 3). The number of grains per spike was the only yield component that was significantly affected by N fertilization, although marginally, since it was not detected by the means test. No significant G × N interaction was observed for GY or any of the yield components.

In contrast, during the 2018/19 season, there were no significant effects of nitrogen fertilizer levels on GY and the agronomic parameters, with the exception of TGW, which slightly increased as nitrogen fertilization rose. By contrast, genotypic differences existed for GY and all the different agronomic yield parameters, except for BDW (Table 3). Soberbio had the highest yield but only differed statistically from Hondia and Benchmark. No significant G × N interaction was observed for GY or any of the yield components.

For the 2017/18 season, the KWS Lili variety showed the highest number of grains per spike within each nitrogen fertilizer level, differing statistically from all the other cultivars, except Benchmark, JB-Diego, and Hondia under the N130 treatment, Bologna, Julius, Henrik, and CH-Nara under the N50 treatment, and Bologna, Julius Chambo, and Soberbio under the N100 treatment (Supplementary Table 3). Bologna had the highest density of spikes (spikes m^−2^) when assayed during the two crop-growing seasons, but while this parameter was statistically different from Hondia alone during 2017/18, only Chambo and Soberbio showed a similar performance to Bologna in 2018/19. Hondia showed the highest TWG in 2017/18, with only Chambo, Julius, and Henrik presenting statistically similar values. In 2018/19, Hondia had the highest TWG under the N50 treatment but was only statistically different from CH-Nara, Bologna, and KWS Siskin, whereas no statistical differences were observed between genotypes under the N100 treatment, and under N130, Bennington showed the best absolute value, but this was only significantly different from Bologna.

### Effect of N Fertilization, Genotype, and Crop Season on Phenological Stages

The phenology of the wheat genotypes tested greatly varied over the two growing seasons and across cultivars. The effects of the year, genotype, and genotype × year interaction were significant for all traits, except the year effect for the period between the beginning of stem elongation and middle grain filling and the genotype effect for the number of days from sowing to tillering (Supplementary Table 4). While the genotypes originating from more northern European countries showed late phenology, the varieties from southern Europe (Bologna, Soberbio, and Chambo) presented earlier phenology (Table 4).

**Table 4:** Summary of the analysis of variance (ANOVA) for nitrogen fertilization (N) and genotypic (G) effects on phenology (number of days from sowing to reach each of the phenological stages (upper part of the table) and mean values across cultivars with each stage (lower part of the table).

Traits	2017/18			2018/19		
	Level of significance (ANOVA)					
	N	G	N × G	N	G	N × G
Tillering	0.032^∗^	0.721^ns^	0.59^ns^	0.344^ns^	0.009^∗∗^	0.775^ns^
Stem elongation	0.834^ns^	0.001^∗∗∗^	0.83^ns^	0.068^ns^	0.000^∗∗∗^	0.000^∗∗∗^
Booting	0.530^ns^	0.000^∗∗∗^	0.39^ns^	0.956^ns^	0.000^∗∗∗^	0.727^ns^
Heading	0.149^ns^	0.000^∗∗∗^	0.23^ns^	0.486^ns^	0.000^∗∗∗^	0.245^ns^
Anthesis	0.400^ns^	0.000^∗∗∗^	0.04^*^	0.301^ns^	0.000^∗∗∗^	0.478^ns^
Middle grain filling	0.893^ns^	0.000^∗∗∗^	0.41^ns^	0.384^ns^	0.000^∗∗∗^	0.719^ns^
PEG	0.844^ns^	0.145^ns^	0.73^ns^	0.129^ns^	0.000^∗∗∗^	0.000^∗∗∗^
	**Significance of the mean ± standard deviation of nitrogen fertilization levels**					
	**N50**	**N100**	**N130**	**N50**	**N100**	**N130**
Tillering	114 ± 5a	111 ± 3b	113 ± 4ab	97 ± 6a	98 ± 6a	99 ± 6a
Stem elongation	137 ± 7a	139 ± 6a	138 ± 6a	126 ± 7a	126 ± 7a	126 ± 7a
Booting	197 ± 6a	197 ± 5a	197 ± 5a	162 ± 4a	162 ± 5a	162 ± 5a
Heading	203 ± 6a	203 ± 5a	203 ± 6a	169 ± 4a	169 ± 4a	169 ± 5a
Anthesis	211 ± 4a	212 ± 5a	211 ± 4a	172 ± 3a	172 ± 3a	172 ± 3a
Middle grain filling	227 ± 5a	227 ± 5a	228 ± 5a	192 ± 5a	193 ± 2a	193 ± 1a
PEG	90 ± 7a	89 ± 5a	90 ± 7a	66 ± 9a	66 ± 6a	67 ± 6a

PEG, period between the beginning of stem elongation and middle grain filling. Phenological stages were determined visually according to the Zadoks scale (Zadoks et al., 1974). Means within the nitrogen levels followed by different letters are significantly different (*p* < 0.05) according to Tukey’s honestly significant difference (HSD) test.

ns Levels of significance for the ANOVA: ns, not significant;

∗ *p* < 0.05;

∗∗ *p* < 0.01;

∗∗∗ *p* < 0.001.

A significant difference due to nitrogen levels among phenological stages was only found for tillering in 2017/18 (Table 4), where tillering was slightly earlier under N100 than the other two treatments. Significant genotype × nitrogen interactions only existed for anthesis in 2017/18 and stem elongation in 2018/19 (Table 4), in the line of the significant genotype by nitrogen by year of interaction for these traits (Supplementary Table 4). From the rest of the phenological traits, only PEG also exhibited a triple interaction.

In 2017/18, Chambo had the earliest crop phenology but did not differ statistically from Bologna at heading, from Bologna, and CH-Nara at anthesis, or from Soberbio and KWS Siskin at middle grain filling (Supplementary Table 5). In 2018/19, Bologna had the earliest phenology, but it did not differ statistically from Soberbio and Chambo at stem elongation, booting, and anthesis, from Chambo at heading, or from Soberbio, Chambo, and CH-Nara at middle grain filling. Furthermore, when the duration of the reproductive development of the genotypes was evaluated (according to the difference in days between middle grain filling and the beginning of stem elongation) only in 2018/19, differences between the genotypes and the varieties of southern Europe (Bologna, Chambo, and Soberbio) showed those with the longest reproductive period, even though the total duration of their development from sowing to mid grain filling was slightly shorter than the rest of the genotypes.

### Effect of N Fertilization, a Genotype, and Crop Season on Vegetation Indices

The performance of the vegetation indices in assessing differences between the genotypes over the two growing seasons was evaluated (Table 5). Due to the lack of a tight matching across crop seasons in the phenological stages, where vegetation indices were measured, the effect of the year (through the three-way ANOVA) was not analyzed in spite of clear evidence in the values were observed between years. In this case, vegetation indices presented significant differences between genotypes for all the measurement dates, with the exception of GGA3 (measured during tillering) in 2018/19. The nitrogen effect was significant in 2017/18 for most of the dates of measurement, except the GGA measurements during tillering and the first NDVI measurement during stem elongation, while, in 2018/19, no differences were observed for any of the data. CH-Nara exhibited consistently lower values for these indices, regardless of the level of fertilization or season, while, in 2017/18, the highest values were mainly associated with JB Diego under N50 and N100 and KWS Siskin under N130. During 2018/19, Soberbio had the highest values until the beginning of stem elongation, but, after this stage, Julius had the highest values for these indices (Supplementary Tables 6 and 7).

**Table 5:** Summary of the analysis of variance (ANOVA) for nitrogen fertilization (N) and genotypic (G) effects on the NDVI (normalized difference vegetation index) and GGA (greener area), and the mean values across nitrogen fertilization levels for these vegetation indices.

Phenological stages	2017/18						
	Level of significance (ANOVA)				Mean ± standard deviation of nitrogen levels		
	Traits	N	G	N × G	N50	N100	N130
Tillering	GGA1	0.826^ns^	0.000^∗∗∗^	0.75^ns^	0.03 ± 0.02a	0.03 ± 0.02a	0.03 ± 0.02a
	GGA2	0.559^ns^	0.000^∗∗∗^	0.29^ns^	0.14 ± 0.08a	0.15 ± 0.08a	0.13 ± 0.07a
Stem elongation	GGA3	0.014^∗^	0.000^∗∗∗^	0.23^ns^	0.42 ± 0.12b	0.54 ± 0.12a	0.50 ± 0.11a
	NDVI1	0.174^ns^	0.000^∗∗∗^	0.39^ns^	0.48 ± 0.09a	0.50 ± 0.08a	0.48 ± 0.07a
	NDVI2	0.049^∗^	0.000^∗∗∗^	0.16^ns^	0.62 ± 0.10b	0.68 ± 0.08a	0.64 ± 0.09ab
Booting	GGA4	0.015^∗^	0.000^∗∗∗^	0.01^∗∗^	0.67 ± 0.08b	0.75 ± 0.07a	0.74 ± 0.09a
	NDVI3	0.033^∗^	0.000^∗∗∗^	0.16^ns^	0.57 ± 0.08b	0.65 ± 0.07a	0.64 ± 0.07a
Anthesis	GGA5	0.000^∗∗∗^	0.000^∗∗∗^	0.01^∗∗^	0.74 ± 0.09b	0.81 ± 0.08a	0.81 ± 0.07a
	NDVI4	0.022^∗^	0.000^∗∗∗^	0.17^ns^	0.68 ± 0.07b	0.72 ± 0.04a	0.71 ± 0.05a
Grain filling	GGA6	0.015^∗^	0.000^∗∗∗^	0.49^ns^	0.47 ± 0.15b	0.57 ± 0.17a	0.57 ± 0.16a
	NDVI5	0.014^∗^	0.000^∗∗∗^	0.29^ns^	0.58 ± 0.07b	0.63 ± 0.07a	0.64 ± 0.06a
	**2018/19**						
3 leaves	GGA1	0.544^ns^	0.000^∗∗∗^	0.548^ns^	0.02 ± 0.00a	0.02 ± 0.01a	0.01 ± 0.00a
Tillering	GGA2	0.755^ns^	0.007^∗∗^	0.965^ns^	0.19 ± 0.06a	0.20 ± 0.09a	0.17 ± 0.07a
	GGA3	0.924^ns^	0.045^∗^	1.000^ns^	0.46 ± 0.10a	0.47 ± 0.11a	0.44 ± 0.12a
	NDVI1	0.679^ns^	0.000^∗∗∗^	0.985^ns^	0.53 ± 0.09a	0.52 ± 0.10a	0.50 ± 0.11a
Stem elongation	GGA4	0.920^ns^	0.002^∗∗^	0.987^ns^	0.63 ± 0.10a	0.62 ± 0.09a	0.61 ± 0.13a
	GGA5	0.566^ns^	0.000^∗∗∗^	0.792^ns^	0.72 ± 0.08a	0.74 ± 0.07a	0.76 ± 0.09a
	NDVI2	0.808^ns^	0.000^∗∗^	0.915^ns^	0.61 ± 0.07a	0.61 ± 0.07a	0.60 ± 0.08a
	NDVI3	0.821^ns^	0.000^∗∗∗^	0.668^ns^	0.70 ± 0.06a	0.69 ± 0.06a	0.68 ± 0.06a
Booting	GGA6	0.852^ns^	0.000^∗∗∗^	0.957^ns^	0.51 ± 0.12a	0.52 ± 0.12a	0.54 ± 0.10a
	NDVI4	0.997^ns^	0.000^∗∗∗^	0.822^ns^	0.52 ± 0.08a	0.52 ± 0.07a	0.52 ± 0.07a
Heading	GGA7	0.869^ns^	0.000^∗∗∗^	0.912^ns^	0.40 ± 0.12a	0.41 ± 0.11a	0.42 ± 0.10a
	NDVI5	0.986^ns^	0.001^∗∗∗^	0.968^ns^	0.44 ± 0.08a	0.44 ± 0.07a	0.44 ± 0.06a
Anthesis	GGA8	0.685^ns^	0.000^∗∗∗^	0.840^ns^	0.19 ± 0.09a	0.19 ± 0.10a	0.21 ± 0.09a
	GGA9	0.720^ns^	0.000^∗∗∗^	0.895^ns^	0.28 ± 0.27a	0.31 ± 0.28a	0.33 ± 0.26a
	NDVI6	0.676^ns^	0.012^∗^	0.998^ns^	0.33 ± 0.05a	0.32 ± 0.05a	0.32 ± 0.05a
Grain filling	GGA10	0.756^ns^	0.000^∗∗∗^	0.846^ns^	0.23 ± 0.26a	0.25 ± 0.33a	0.22 ± 0.27a

Means of the nitrogen levels followed by different letters are significantly different (*p* < 0.05) according to Tukey’s honestly significant difference (HSD) test.

ns Levels of significance for the ANOVA: ns, not significant;

∗ *p* < 0.05;

∗∗ *p* < 0.01;

∗∗∗ *p* < 0.001.

### Correlations Between Yield Components and Grain Yield

The genetic correlations of GY with the yield components across the 12 cultivars were examined for each growing season, combining data of all the three nitrogen fertilization levels (Table 6). For 2017/18, the crop season with the highest yield, there were no relationships between any of the yield components and GY. In 2018/19, the crop season with the lowest yield, there was a strong positive correlation between GY and ^—^SM and GS. These same results can also be observed through phenotypic correlations that considered the 108 individual plots (Supplementary Table 8). In addition, genetic (Table 6) and phenotypic (Supplementary Table 8) correlations of the aerial dry biomass with grain yield were not significant.

**Table 6:** Genetic correlation coefficients of grain yield with yield components.

Season	TGW	BDW	SM	GS
2017/18	0.10^ns^	0.06^ns^	0.15^ns^	0.31^ns^
2018/19	−0.37^ns^	0.0^ns^	0.78^∗∗^	0.93^∗∗^

GY, grain yield; TGW, thousand grain weight; BDW, biomass dry weight; SM, spikes m^−2^; GS, grains spike^−1^.

ns Not significant;

∗∗ *p* < 0.01.

### Genetic Correlations Between Phenological Stages and Grain Yield

**Figure 3:**
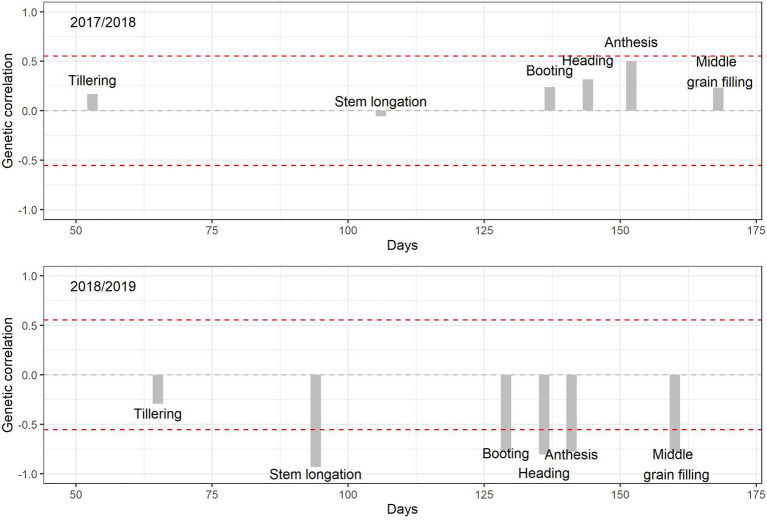
Genetic correlation coefficients between grain yield (GY) and the time taken to reach the different phenological stages from sowing. The red dashed lines represent the threshold significance levels (*p* < 0.05). The *x*-axis represents the number of days from the beginning of the 2017/18 and 2018/19 seasons.

**Figure 4:**
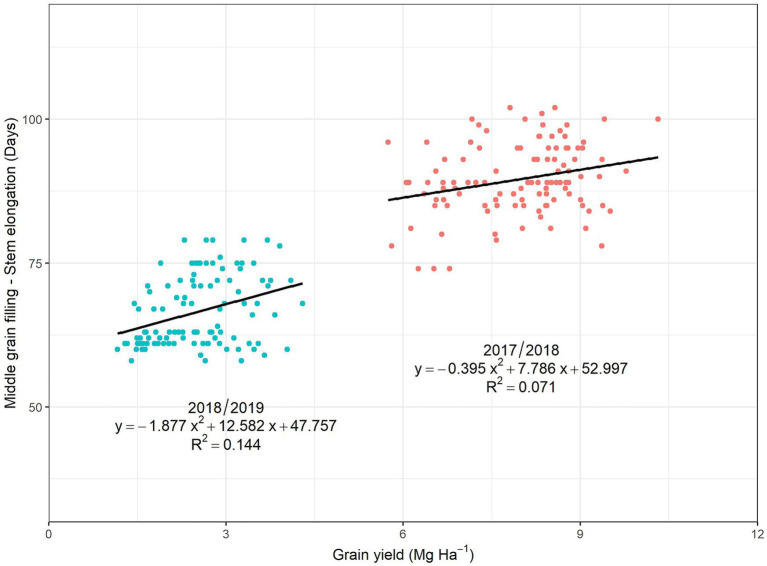
Relationships of grain yield with the number of days between middle grain filling and the beginning of the stem elongation, measured in the 2017/18 (blue points) and 2018/19 (red points) crop seasons. For each crop season, correlations were calculated across the 108 plots.

The genetic correlation across the 12 cultivars between GY and the number of days to reach the different phenological stages after sowing were calculated for each of the two growing seasons by pooling data from the three levels of nitrogen fertilization (Figure 3). During the 2017/18 season, the relationship of the number of days to achieve tillering and stem elongation with GY was very weak; the strength of the relationship increased through booting and heading, almost reaching significance (*p* < 0.05) at anthesis and decreasing during middle grain filling. On the other hand, for the second season, the correlations were negative and significant in all phenological stages, except for tillering. The phenotypic correlations for the two seasons, evaluated by considering the 108 plots of the experiment (Supplementary Table 8), showed practically the same pattern as the corresponding genetic correlations. On the other hand, when the relationships of GY with the number of days between middle grain filling and the beginning of stem elongation were evaluated, the phenotypic correlations (Figure 4) were positive and significant, following the same pattern in both crop seasons. In the same sense, the genetic correlations were high and positive, 0.78 in 2017/28 and 0.90 in 2018/19.

### Genetic Correlations Between Vegetation Indices and Grain Yield

**Figure 5:**
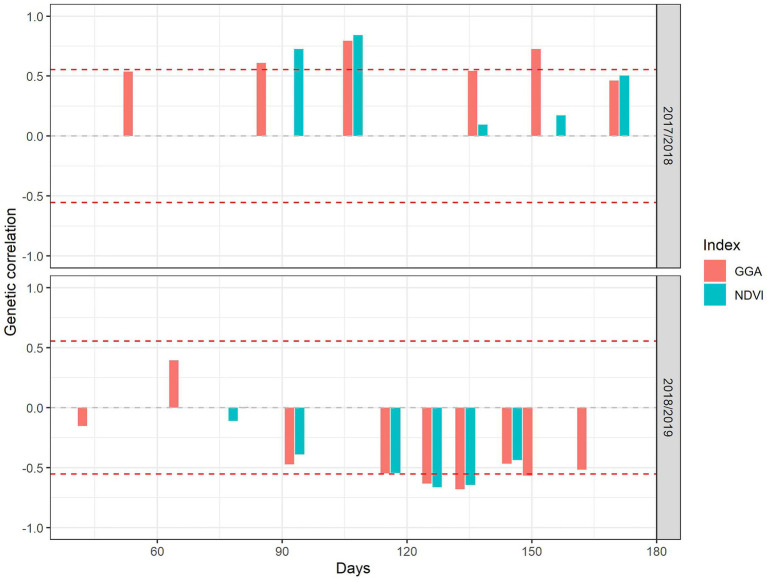
The genetic correlation coefficient of grain yield with greener area (GGA) and the normalized difference vegetation index (NDVI). The red dashed lines represent the threshold significance levels (*p* < 0.05). The horizontal axis represents the number of days from the beginning of the 2017/18 and 2018/19 seasons.

Genetic correlations between GY and both the NDVI and the GGA were calculated for each crop season, across the 12 cultivars, and by combining data from all three nitrogen fertilizer levels (Figure 5). In 2017/18, the NDVI and GGA correlated positively with GY throughout the growing season. The correlations obtained for the NDVI were high around the stem elongation period, decreased between booting and anthesis, and increased again during grain filling. Nevertheless, compared with the NDVI, GGA had a consistently stronger correlation with GY, particularly during heading and anthesis. In contrast, phenotypic correlations that considered the 108 plots in the experiment showed a similar pattern of positive correlations, but, in this case, the NDVI and GGA correlations with GY were similar (Supplementary Table 8).

In 2018/19, the genetic correlations of vegetation indices with GY were negative, with the exception of the two GGA measurements during tillering. From stem elongation onward, the values of the correlation coefficients stabilized close to −0.60. However, phenotypic correlations that considered the 108 experimental plots (Supplementary Table 8) were, in general, very weak, with the only significant and positive correlations for NDVI being between booting and anthesis and for GGA being between anthesis and grain filling.

### Principal Component Analysis

**Figure 6:**
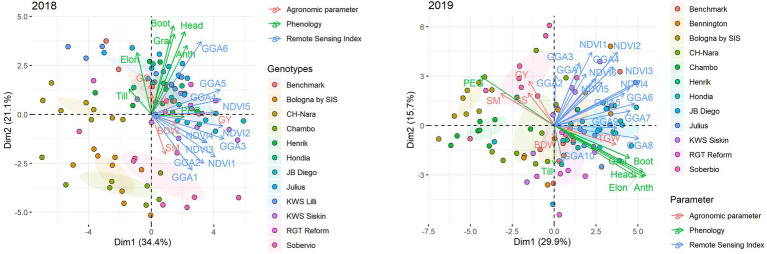
The principal componenty analysis (PCA) of grain yield, agronomic yield components, and phenological traits, and vegetation indices measured at different stages of the crop in the 2017/18 (**left figure**) and 2018/19 (**right figure**) growing seasons. Traits included in the PCA are GY, grain yield; TGW, thousand grain weight; BDW, biomass dry weight; SM, spikes m^−2^/number of spikes per square meter; and GS, grains spike^−1^. Phenological stages: Till, tillering; Elon, stem elongation; Boot, booting; Head, heading; Anth, anthesis; Grai, middle grain filling; and PEG, period between stem elongation and middle grain filling. Vegetation indices: NDVI: the normalized difference vegetation index; GGA, the greener area. GGA and NDVI evaluations were carried out at the following stages: GGA (2017/18): 1, 2 (tillering), 3 (stem elongation), 4 (booting), 5 (anthesis), and 6 (grain filling). NDVI (2017/18): 1, 2 (stem elongation), 3 (booting), 4 (anthesis), and 5 (grain filling). GGA (2018/19): 1 (three expanded leaves) 2, 3 (tillering), 4, 5 (stem elongation), 6 (booting), 7 (heading), 8, 9 (anthesis), and 10 (grain filling). NDVI (2018/19): 1 (tillering), 2, 3 (stem elongation), 4 (booting), 5 (heading), and 6 (grain filling).

To summarize the relationships existing between GY, agronomic components, phenology, and the vegetation indices measured at different stages, a PCA was performed, including all these traits (Figure 6). The first two principal components of the PCA explained 58.3 and 46.5% of the observed variability in 2017/18 and 2018/19, respectively. In 2017/18, GGA3, GGA4, GGA5, NDVI2, NDVI3, NDVI5 were the traits most closely related to GY. There was a slight separation between genotypes in the 2017/18 growing season, and these were directly related to the phenology. A group formed by Benchmark, Henrik, Hondria, JB Diego, Julius, KWS Lili, KWS Siskin, and RGT Reform, which are all genotypes from central and northern Europe and with longer phenological cycles, was located in the upper right side of the figure. Another group, at the bottom of the figure, formed by Soberbio, Bologna, and Chambo (cultivars from southern Europe), was placed further away from the other genotypes and negatively related to phenological traits, with Bologna and Chambo both exhibiting the lowest GYs. The CH-Nara variety was the only one placed between these two groups, with intermediate phenology and low GY. In 2018/19, the traits most closely related to GY were yield, ^—^SM, and GS, along with the GGA measured during tillering and the number of days between stem elongation and middle grain filling (PEG). The duration of phenological stages was negatively related to GY and closely associated with each other. Overall, the vegetation indices were distributed between GY and the phenological traits, with the vegetation indices measured early in the crop cycle being closer to GY, while the final evaluations were closer to the phenological traits. There was no clear separation between genotypes in the 2018/19 growing season but only small displacement toward the GY side for the most productive genotypes with shorter phenology (Soberbio, Bologna, Chambo, and CH-Nara). Of these, all exhibited the lowest phenological cycles in both seasons. Already, the cultivars KWS Siskin and Henrik from central/northern Europe which performed well during the two growing seasons exhibited intermediate phenological cycles.

## Discussion

In the 2018/19 crop season, there was a substantial reduction in GY among the varieties, compared with the previous 2017/18 crop season. This was due to an extremely dry year, with low precipitation during the winter and the spring, particularly from January to April (Figure 1). In addition to the lack of rain, the temperature relative to the previous season was also higher: 3.5°C during winter and 1.5°C during spring, which caused an increase in the evaporative demand (Figure 1). In fact, the total accumulated rainfall and potential evapotranspiration from the beginning of January to mid-June were 420.6 and 459.6 mm for 2017/18 and 121.3 and 537.9 mm for 2018/19, respectively, while average values for this location during the last 10 years were 214.1 and 580.3 mm, respectively.^2^ The accumulated precipitation from sowing to anthesis and from anthesis to maturity was 393.95 and 92.60 mm for 2017/18 and 186.08 and 30.60 mm for 2018/19, respectively; this represents a reduction of 53% before anthesis and of 67% after anthesis in 2018/19. Under Mediterranean conditions, water stress and high temperatures are common, and these cause drought stress (Soriano et al., 2018). During 2018/19, drought was already present during the early crop stages, as shown by the lower NDVI and GGA values already recorded at the three-leaf stage and at the beginning of stem elongation, and unlike the conditions of 2017/18 (Supplementary Tables 6 and 7). As a consequence, the yield achieved during 2018/19 was low (average across genotypes and treatments was 2.52 Mg ha^−1^), whereas the GY average for 2017/2018 was 7.95 Mg ha^−1^. In fact, these 2 years were among the most and least productive during the periods 2004 to 2020 in terms of wheat yield at both farmer fields and experimental trials of Castilla y León, the Spanish region where this experiment was carried out (Supplementary Figure 1). Even under lower N fertilization conditions (N50), average production was above 5.89 Mg ha^−1^, corresponding to the medium–high production conditions achieved under wet Mediterranean environments (Senapati and Semenov, 2020).

### Effect of N and Climatic Conditions on Crop Growth and Yield

Adequate nitrogen fertilization represents an essential factor to maximize crop production (Gastal et al., 2015; Barber et al., 2017). However, excess applications can surpass the capacity of the plant to absorb and store N, leading to losses *via* leaching during rain or irrigation (Good and Beatty, 2011; Gastal et al., 2015). Moreover, excess N fertilization may negatively affect yield through different mechanisms. For example, it can increase the rate of lodging (Gastal et al., 2015; Kefauver et al., 2017), cause excessive tiller production (Ravier et al., 2017), or risk haying-off, which may occur under Mediterranean rainfed conditions. The latter is attributed to a negative yield response due to exhaustion of soil water, which is caused by vigorous vegetative growth being stimulated by high soil nitrogen levels (van Herwaarden et al., 1998; Araus et al., 2013). In our results, there were no significant differences between N100 and N130 irrespective of the crop season (even when a tendency was present in the wetter crop season for a lower yield and spike density under N130), while N50 had a significant, albeit slight, decrease in GY in the wetter crop season. In agreement with this, low fertilization in the wet crop season caused a decrease in the vegetation index values during most of the crop cycle, except in the earliest and latest stages. Nevertheless, no statistical differences were observed for the yield components and biomass at anthesis between the extreme nitrogen fertilization regimes in both crop seasons. Due to adverse weather conditions in 2018/19, the effects of nitrogen fertilization on the vegetation indices and grain yield were even less evident, but no haying-off occurred. Although it is possible to associate the effects of both water and nitrogen deficits (Cossani and Sadras, 2018), in conditions of severe water stress, nitrogen absorption is limited by water, and, therefore, the availability of N is a less important factor for production than water constraints (Abeledo et al., 2008; Eitel et al., 2008). Although N deficiency may affect plant growth (Barber et al., 2017; Ravier et al., 2017), leading to lower rates of biomass accumulation (Gastal et al., 2015), our results clearly indicate that the actual recommended rates of nitrogen fertilization in the region are excessive, even under well-watered conditions, and a strong reduction in top fertilization had no consequences (at least in the short term) in terms of grain yield. In this sense, we can still point out that the differences found in the N50 treatment, compared with the other two nitrogen treatments, were due to the fact that this treatment received its total amount of nitrogen fertilizer in the first application, which may have led to a possible depletion of the nitrogen reserves of the soil during the second part of the cycle, unlike other treatments that receive a second top-dressing applications of nitrogen. A lack of effect (and even a positive effect) associated with reductions in nitrogen fertilization has been reported before for the Mediterranean continental conditions of Spain (Araus et al., 2013). In addition, nitrogen fertilization did not affect crop phenology. While inadequate nitrogen may accelerate wheat phenology, our results did not show such an effect, which further supports the hypothesis that a 50% reduction in the recommended nitrogen fertilization rates does not cause significant yield limitations.

### Correlations Between Yield Components and Grain Yield

In the favorable conditions of the 2017/18 growing season, there were no relationships between the yield components with GY. The absence of such correlations is often reported under highly productive growing conditions due to inadequate genotypic variability (Slafer et al., 2014; Ferrante et al., 2017; Fernandez-Gallego et al., 2018) or the compensatory responses of the different agronomic components that determine yield (Slafer et al., 2014; Ferrante et al., 2017). On the other hand, strong correlations between the GY and the agronomic components are often found under stress conditions (Chairi et al., 2020). In 2018/19, a larger GY was attributed to a greater number of spikes m^−2^ and GS, the two components that determine the number of kernels per unit ground area, which is usually the main agronomic determinant of grain yield. Accordingly, drought stress in this season was already present during the first part of the crop cycle (Figure 1) and, therefore, was strongly affected the number of ^—^SM and GS, which are the first two yield components formed during the crop cycle (Simane et al., 1993; Slafer et al., 2014). Even TGW was much lower in the second crop season compared with the first, which indicates that drought stress was also present during grain filling.

### Correlations Between Phenology and Grain Yield

In the first season of evaluation, there was a tendency for late genotypes, particularly in terms of the date of anthesis, to be related to higher GY, although the correlations were not significant. Late anthesis can help to maximize the production of pre-anthesis biomass, which contributes to a higher yield (Senapati and Semenov, 2020) and agrees with the positive relationships between GGA at anthesis and GY found in the 2017/18 season. In general, under good agronomic conditions, a longer phenological cycle can guarantee not only a greater production of pre-anthesis biomass but also the maximization of yield components, namely the number of spikes, production of florets, grains per spike, and grain weight (Senapati and Semenov, 2020). This can be proven by the high and significant genetic correlation of the period between the beginning of the stem elongation and middle grain filling with GY. Moreover, better grain filling may be also associated with stay-green (Christopher et al., 2016; Gracia-Romero et al., 2019), which has proven its positive effect even under high yielding conditions of the UK (Carmo-Silva et al., 2017). Nevertheless, the relationship between total crop duration (days from sowing to middle grain filling) and GY was weaker than between anthesis time and GY.

In the second crop season (2018/19), the earliest varieties were strongly related to higher GY. Early anthesis in wheat is considered a very common mechanism to escape drought during anthesis (Araus, 2002; Savin et al., 2015; Shavrukov et al., 2017). Therefore, even if drought had already been present from the early vegetative phases (Figure 1), escaping drought was a positive mechanism. In the case of durum wheat, a short period of booting has been identified as an adaptive evolutionary mechanism to escape high temperatures and late-season drought in warm Mediterranean regions (Isidro et al., 2011; Soriano et al., 2018). Thus, even if the varieties with earlier phenology were the most productive, their genetic correlation of the time period between the beginning of stem elongation and middle grain filling was positive and significantly correlated with GY. Rapid progression to the stem elongation stage may have guaranteed these genotypes a greater capacity to develop in the subsequent phases. Thus, these earlier genotypes were more likely to develop a higher number of spikes, with more grains per spike and subsequently higher GY (Soriano et al., 2018). In this study, and for both of the crop seasons, the southern varieties also developed a larger number of spikes than most of the northern cultivars. On the other hand, later genotypes are more exposed to suffering drought under Mediterranean conditions, particularly coinciding with the last part of the crop cycle (Soriano et al., 2018; Senapati and Semenov, 2020). Hunt et al. (2019) have reported that earlier sowing of long-cycle (i.e., winter) genotypes may favor productivity under the Mediterranean conditions of Australia. However, such a strategy is not always feasible to implement, since it depends on the environmental conditions (basically, the pattern of rainfall during late summer, spring) which are particularly variable under Mediterranean conditions. In our study, the planting date for the second growing season 2018–19 s cycle was relatively late due to climate conditions.

### Correlations Between Vegetation Indices and Grain Yield

In 2017/18, the best genetic correlations between GGA and GY were for measurements of the vegetation index during intermediate stages of the crop cycle, between the booting and anthesis phases. Indeed, heading and anthesis have been indicated as the best phenological stages for remote sensing assessment of grain yield (Yousfi et al., 2016; Fernandez-Gallego et al., 2019). This period comprises the most critical phases that determine the number and the weight of grains in wheat (Shavrukov et al., 2017). However, in the case of NDVI measurements during this period, their correlation with GY was very low, probably reflecting the nature of this index, which saturates at values beyond 0.65 (Marti et al., 2007; Duan et al., 2017). This may be the case under good agronomic conditions when the vegetation is dense, usually coinciding with the reproductive period (Gracia-Romero et al., 2019). In support of this, in the best of the two seasons, which was characterized by large canopies during the reproductive stage, the best correlation of NDVI with GY was attained during the first part of the crop cycle at stem elongation. Marti et al. (2007) reported that assessment of the NDVI at the beginning of stem elongation could predict wheat production. In the case of GGA, because it considers only the greenest area of the plant, it is not as prone to saturation as the NDVI (Gracia-Romero et al., 2019), and, therefore, compared with the NDVI, it presents advantages in detecting genotypic differences in the green canopy during the reproductive stages that may eventually translate to a better correlation of this index with GY. This would explain the high correlation coefficients found at anthesis. During grain filling, the green area decreased, which is associated with crop senescence. In this sense, during the best crop season, the relationship between the NDVI measured during grain filling and GY increased to some extent relative to the very low values recorded at anthesis, indicating a trend for better production in genotypes with prolonged green canopies (i.e., stay-green). According to Christopher et al. (2016) and Gracia-Romero et al. (2019), vegetation indices assessed throughout grain filling may be suitable for detecting more productive genotypes because they allow the identification of genotypes that stay green for extended periods. Stay-green is related to a longer period of active photosynthesis and, consequently, maintenance of carbon assimilation, ensuring that the grain mass is maximized (Lopes and Reynolds, 2012). A longer stay-green has been associated with genetic advances in the GY of wheat under the highly productive conditions of the UK (Carmo-Silva et al., 2017). Although it was not the main factor, these findings led us to believe that stay-green during grain filling helped to improve genotypic performance under the good agronomic conditions of the 2017/18 crop season.

In the 2018/19 season, all genetic correlations between vegetation indices and GY after stem elongation were negative. These results are also supported by the phenotypic correlations between the 108 individual plots. Negative correlations of the GGA and NDVI measured at anthesis with GY have been reported previously in wheat under Mediterranean conditions (Rezzouk et al., 2020). The relationships of GGA assessed earlier during the crop cycle (at tillering) to GY were positive. A positive relationship was also found at the same phenological stage in the wet growing season of 2017/18. This suggests that the genotypes tended to behave the same way in terms of crop establishment during the early stages of both crop seasons. However, in 2018/19, the occurrence of a severe drought during this growing season implied that a larger canopy later in the crop cycle was a negative trait in terms of GY, probably due to the occurrence of water stress that was associated with more rapid exhaustion of soil water. The negative effect of a large canopy under drought conditions, exhausting water resources and then negatively affecting GY, has been extensively reported in wheat under Mediterranean conditions (Isidro et al., 2011; Soriano et al., 2018).

### Defining the Ideotype: PCA

The PCA analysis indicated that, in the absence of drought (2017/18 crop season), greater green biomass throughout the crop cycle (as assessed by the vegetation indices GGA and NDVI) was the trait best associated with GY, especially measurements not only during stem elongation but also during the reproductive stage, and related to the presence of stay-green during grain filling. Quick crop establishment, covering the soil earlier, optimizes canopy photosynthesis while decreasing evaporative losses, therefore contributing to a higher yield (Marti et al., 2007). On the other hand, as discussed earlier, genotypes with larger canopies that cover the soil earlier and maintain a stay-green condition are correlated with a long and stable period of active photosynthesis and consequently maintain carbon assimilation, ensuring that production of yield components is maximized, such as grain number and weight (Marti et al., 2007; Lopes and Reynolds, 2012). In the stress conditions of the 2018/19 crop season, the ^—^SM and GS production traits were most closely correlated with GY. These components were the most affected by a lack of water during the first part of the crop cycle; the consequence of which was a large reduction in the number of kernels per unit ground area, which is the main agronomic determinant of grain yield (Simane et al., 1993; Slafer et al., 2014). Greater numbers of ears with more grains per ear have been considered as adaptive mechanisms for wheat to guarantee grain yield under the frequent stress conditions of the Mediterranean (Araus, 2002; Isidro et al., 2011; Soriano et al., 2018). On the other hand, GY was negatively associated with crop phenology but positively associated with a longer reproductive stage, encompassing the beginning of stem elongation to middle grain filling. Thus, genotypes that reached the reproductive stage (beginning of stem elongation) earlier were more likely to exhibit adequate development during the following stages and consequently establish a greater number of ears with more grains. In contrast, genotypes with a longer vegetative stage and a later date of reproductive phenology depleted the available water resources, with ultimately poorer conditions for the formation of yield components and GY. Moreover, a larger green area during stem elongation (as assessed by GGA) was positively associated with GY, indicating a faster establishment of the most productive varieties in this phase.

In 2017/18, the separation between genotype groups was directly related to phenological characteristics. On the other hand, in 2018/19, the separation between genotype groups was directly related to GY and inversely related to the phenology. The results show that varieties from central and northern Europe belonged to the group with longer phenological cycles, whereas varieties from southern Europe were in the group with the shortest phenological cycles, regardless of the crop season. The varieties from southern Europe were among the best adapted and highest yielding genotypes under drought (crop season 2018/2019). Due to the more limiting conditions found in southern Europe, where low precipitation and high temperatures lead to frequent drought stresses during the reproductive phases of the crop, the best adapted genotypes exhibited an earlier start to the reproductive stage and, in general, a shorter crop cycle (Araus, 2002), along with other traits such as a high frequency of alleles that confer a greater number of spikes and grains for given crop duration (Isidro et al., 2011; Soriano et al., 2018). On the other hand, the favorable climatic conditions in the central and northern European regions with their low incidences of heat and drought stresses implies the need to select for traits that increase yield potentials such as longer crop duration, including delayed anthesis and later maturity, which can result in greater interception of solar energy and grain yield (Senapati and Semenov, 2020).

### Cultivar Resilience: Growth Phenology and Grain Yield

Grain yield and all the yield components assessed, except for crop biomass, exhibited genotypic differences within the two contrasting crop seasons. Moreover, regardless of the crop season, we found no genetic or phenotypic correlations between the aerial biomass and grain yield. In fact, the improvement in wheat over the past 50 years has not been characterized by an increase in biomass (Acreche et al., 2008). The moderate winter temperatures in Mediterranean conditions lead to low wheat tillering ability, in addition to accelerating each stage of development. For this reason, recommended sowing rates (400–500 plants m^−2^) in northwestern Spain to secure a fast and complete ground cover are generally higher than those reported in areas of north-central Europe and United States, with longer growing periods, where maximum yields are normally obtained with sowing density of around 200–300 seeds m^−2^ (Lloveras et al., 2004).

The provenance of the varieties had an effect on their agronomic performance throughout the two contrasting growing seasons. Thus, in the wet season (2017/18), cultivars from central/northern Europe (KWS Siskin, United Kingdom; JB Diego, Ireland; Benchmark, Germany; RGT Reform, Germany; Henrik, Belgium) were the most productive, while the southern European cultivars were placed in the middle (Soberbio and Chambo, Spain) or were the least productive (Bologna, Italy). In contrast, in the dry growing season (2018/19), one of the three southern European cultivars (Soberbio) was placed first in terms of yield, while the other two occupied the fourth (Chambo) and fifth (Bologna) positions. Interestingly, two cultivars from central/northern Europe performed well during the two growing seasons, with KWS Siskin being the first and second most productive, during the two consecutive seasons, respectively, and Henrik being the fifth and the third most productive, respectively. In 2017/18, Soberbio (221 days), Chambo (220 days), and Bologna (223 days) had the shortest crop duration (from sowing to middle grain filling), while KWS Siskin (228 days); Benchmark, RGT Reform, and Henrik (all three with 229 days); and JB Diego (230 days), exhibited intermediate to long duration. In 2018/19, Soberbio, Chambo, and Bologna had the shortest cycle (191 days), while the cycles of the central/northern European genotypes were slightly longer (193–194 days). Therefore, the differences in the number of days among the cultivars were minor. However, differences in the interval between the beginning of stem elongation and middle grain filling were more evident, with development in Bologna (76 days) and Soberbio and Chambo (72 days) being 10 or more days longer than in RGT Reform, JB Diego, and Benchmark. Interestingly, KWS Siskin (69 days) and Henrik (66 days) exhibited intermediate values. Phenology adjustment has been reported as one of the main traits that confer agronomic adaptation under Mediterranean conditions (Slafer, 1994; Araus, 2002; Marti et al., 2007), and it has usually been related to earliness in terms of time to heading and anthesis. However, the present results suggest that the duration of the reproductive period is even more important, where the components determining yield are defined (Figure 3). In fact, the results for 2018/19 suggest that ear density and grains per ear (the first two yield components defined during the crop cycle), rather than TGW, are the main determinants of grain yield across cultivars (Table 6). In fact, the three cultivars from southern Europe were also characterized by a greater number of ears per area, which is in line with reports on an adaptive mechanism for the frequent stress conditions of southern Europe (Araus, 2002; Isidro et al., 2011; Soriano et al., 2018). Under Mediterranean conditions, the only feasible way to extend the reproductive period is through an earlier start to the reproductive stage, which is assessed *via* the date of stem elongation (Slafer, 1994; Araus, 2002; Marti et al., 2007). In fact, in our study, the date of commencement of stem elongation was the phenological trait that best correlated (negatively) with grain yield in 2018/19 (Figure 2). Therefore, an earlier date for stem elongation was also associated with a good adaptation to Mediterranean dry conditions. Thus, during 2018/19, the values for Bologna (115 days) and Soberbio and Chambo (119) were at least 2 weeks earlier than RGT Reform (133 days), JB Diego (132), and Benchmark (131 days). However, KWS Siskin (124) and even Henrik (127) exhibited intermediate values.

On the other hand, a trend toward greater precocity, mainly during the reproductive stages, has led not only to shorter phenology (e.g., shorter heading and anthesis times) but also to an overall reduction in the crop cycle duration (Slafer, 1994; Araus, 2002; Langer et al., 2014; Soriano et al., 2018). Although it is known that drought and heat accelerate anthesis (Shavrukov et al., 2017), which agrees with the shorter phenologies of the cultivars in the second crop season (Supplementary Table 5), genotypic differences still existed in this growing season. In fact, southern European varieties were associated with a lower GGA during the last measurements (end of grain filling), indicating that these varieties are the first to start leaf senescence due to their shorter phenological cycle. This essentially corresponds with the overall reduction in the crop cycle duration that has been associated with breeding for Mediterranean conditions (Araus, 2002; Langer et al., 2014; Soriano et al., 2018), which may also limit the potential yield achievable in a wet growing season. Indeed, in the 2017/18 season, where there was an absence of drought, some of the central/northern European cultivars outyielded the cultivars from southern Europe. Interestingly, the CH-Nara cultivar from Switzerland, with crop duration only slightly longer than the southern European cultivars, exhibited the lowest yield in 2017/18 but was placed in the middle (sixth place) in 2018/19.

Besides the above considerations about phenology, differences in growth may also be involved. This is exemplified when considering the best cultivars from central/northern and southern Europe, namely, KWS Siskin and Soberbio, respectively. These two cultivars showed high vegetation indices, with KWS Siskin, generally exhibiting the highest values in both seasons, while Soberbio had the highest values during the vegetative phases in 2018/19. Fast soil coverage after plant emergence is also a trait selected for Mediterranean conditions that aim to minimize evaporation from the soil while optimizing transpiration (Marti et al., 2007).

## Conclusion

Our results provide clues not just on the traits associated with the genetic advance of bread wheat varieties across Europe but also, more importantly, on reports on the comparative performance under contrasting Mediterranean conditions.

While the lack of drought stress in central/northern Europe has led to optimization of traits, such as a longer crop cycle, including delayed time to anthesis and later maturity, the frequency of water and heat stress in southern European regions has led to increases in the reproductive period with regard to the total crop duration, thus increasing the number of ears per area in order to guarantee grain yield. As a consequence, greater green biomass and its maintenance during grain filling (i.e., stay-green) are traits that determine higher yields in wetter crop seasons, while earliness for the start of the reproductive cycle (i.e., shorter times to stem elongation) ensures greater numbers of spikes and grains and, therefore, higher yields in dry crop seasons. This occurs mainly when drought stress is long and starts at an early phenological stage. On the other hand, regardless of the water regime, the results support the hypothesis that the recommended doses of nitrogen fertilizer are probably excessive, and a strong reduction in top fertilization had no consequences (at least in the short term) for grain yields.

In view of our results, the best genotypes for future climate change scenarios of increased temperatures and drought in European regions are those with modified phenology, particularly in terms of optimizing the duration of the reproductive stage, together with rapid growth during the early stages of the crop cycle to ensure a greater number of spikes and grains per unit ground area. However, the challenge to breeding programs will be to increase adaptation to drought conditions without penalizing yield potential (Senapati and Semenov, 2020). In this sense, the wide resilience to Mediterranean conditions shown by the genotype KWS Siskin from central/northern Europe (and also, to some extent, by Henrik) opens new avenues for exploration. Interestingly, these genotypes exhibited intermediate phenologies and relatively long reproductive periods and, in the case of KWS Siskin, fast growth was maintained throughout the crop cycle.

## Data Availability Statement

The raw data supporting the conclusions of this article will be made available by the authors, without undue reservation.

## Author Contributions

VL: formal analysis, investigation, writing – original draft, and visualization. AG-R: formal analysis, investigation, and writing – original draft. FR: investigation and writing – review and editing. MD-F, IA-G, and SKe: investigation. SKa: formal analysis, writing – original draft, and review and editing. AA: writing – review and editing. NA: conceptualization, methodology, investigation, resources, writing – review and editing, project administration, and funding acquisition. JLA: conceptualization, methodology, investigation, resources, writing – original draft, review and editing, supervision, project administration, and funding acquisition. All authors contributed to the article and approved the submitted version.

### Conflict of Interest

The authors declare that the research was conducted in the absence of any commercial or financial relationships that could be construed as a potential conflict of interest.

## Figures and Tables

**Table:** 

Term	Definition
GxExm	Interaction of genotype, target environment, and management condition
NDVI	Normalized difference vegetation index
GGA	Greener area
ECOFE	European Consortium for Open Field Experimentation
ET0	Potential evapotranspiration
N100	Optimum nitrogen fertilization recommended in the zone
N50	50% of the optimum N dose recommended in the zone
N130	30% above the optimum
DAS	Days after sowing
N	Nitrogen fertilization
G	Genotypic
Y	Year
GY	Grain yield (Mg ha^−1^)
TGW	Thousand grain weight
BDW	Biomass dry weight
SM	Spikes m^−2^/number of spikes per square meter
GS	Grains spike^−1^
Till	Tillering
Elon	Stem elongation
Boot	Booting
Head	Heading
Ant	Anthesis
Grai	Middle grain filling
PEG	Period between stem elongation and middle grain filling

## Footnotes

^1^https://www.ecofe.eu/

^2^http://www.siar.es

## Supplementary Material

Supplementary Table 1 Soil composition before sowing the two consecutive crop growing seasons (2017/18 and 2018/19), at Zamadueñas Station, Valladolid, (41°41'N, 04°42'W, 700 m altitude), from the Institute Tecnológico Agrario de Castilla y León (ITACyL).

Supplementary Table 2 Summary of the analysis of variance for the year (Y), nitrogen fertilization (N), and genotypic (G) effects and their interactions for GY and yield components. Levels of significance for the ANOVA: ns, not significant; **p* < 0.05; ***p* < 0.01; ****p* < 0.001.

Supplementary Table 3 Means ± standard deviation of genotypes for agronomical yield components and biomass. The means followed by different letters are significantly different (*p* < 0.05), according to Tukey’s honestly significant difference test (HSD).

Supplementary Table 4 Summary of the analysis of variance for the year (Y), nitrogen fertilization (N), and genotypic (G) effects and their interactions on phenology (number of days from sowing to reach each of the phenological stages). Levels of significance for the ANOVA: ns, not significant; **p* < 0.05; ***p* < 0.01; ****p* < 0.001.

Supplementary Table 5 Means ± standard deviation of genotypes for phenological stages in days after sowing. The means followed by different letters are significantly different (*p* < 0.05), according to Tukey’s honestly significant difference test (HSD).

Supplementary Table 6 Means ± standard deviation of genotypes for vegetation indices of 2017/18. The means followed by different letters are significantly different (*p* < 0.05), according to Tukey’s honestly significant difference test (HSD).

Supplementary Table 7 Means ± standard deviation of genotypes for vegetation indices of 2018/19. The means followed by different letters are significantly different (*p* < 0.05), according to Tukey’s honestly significant difference test (HSD).

Supplementary Table 8 Pearson’s correlation coefficient of grain yield with yield components, phenological stages, and the vegetation indices greener area (GGA), and normalized difference vegetation index (NDVI) (*n* = 108).

Supplementary Figure 1 Comparison of the average grain yield of 2 years of evaluation of our experiment (ours), with the yield of commercial cultivars of the farmers (farmers) and experimental trials of new registrated varieties (trials), carried out by ITACyL (Instituto Tecnológico Agrario de Castilla y León) across different environments, between 2004 and 2020, for the region of Castilla y León, Spain.
