# Supplementary material for: Comparative Performance of High-Yielding European Wheat Cultivars Under Contrasting Mediterranean Conditions
Source: Front Plant Sci. 2021 Jun 29;12:687622. doi: 10.3389/fpls.2021.687622 (PMC8276830; doi:10.3389/fpls.2021.687622)
Supplement: Supplementary Table 1 — Soil composition before sowing the two consecutive crop growing seasons (2017/18 and 2018/19), at Zamadueñas Station, Valladolid, (41°41'N, 04°42'W, 700 m altitude), from the Institute Tecnológico Agrario de Castilla y León (ITACyL). [file Data_Sheet_1.docx]

**Supplementary Tables**

**Supplementary Table 1.** Soil composition before sowing the two consecutive crop growing seasons (2017/18 and 2018/19), at Zamadueñas Station, Valladolid, (41° 41'N, 04° 42'W, 700m altitude), from the Institute Tecnológico Agrario de Castilla y León (ITACyL).

| **REFERENCE** | **2017/18** | **2018/19** |
| --- | --- | --- |
| **Silt (%)** | 54 | 15 |
| **Sand (%)** | 25 | 44 |
| **Clay (%)** | 22 | 41 |
| **Texture** | Silty-loam | Loam |
| **Conductivity [(1:5 25ºC) µS/cm]** | 124 | 109 |
| **pH in water (1:5)** | 8.52 | 8.56 |
| **Removable potassium in ammonium acetate (mg K_2_O g^-1^)** | 0.29 | 0.25 |
| **Total carbon (%)** | 0.82 | 0.32 |
| **Organic carbon (dry combustion, %)** | 0.64 | 1.58 |
| **Organic matter (dry combustion, %)** | 1.10 | 2.98 |
| **Carbonates (dry combustion, %)** | 9.80 | 7.56 |
| **Phosphorus (mg P_2_O_5_ Kg^-1^)** | 60 | 62 |
| **Total nitrogen (dry combustion, %)** | 0.139 | 0.057 |

**Supplementary Table 2.** Summary of the analysis of variance for the year (Y), nitrogen fertilization (N), and genotypic (G) effects and their interactions for GY and yield components. Levels of significance for the ANOVA: ns, not significant; *, *P* < 0.05; **, *P* < 0.01; ***, *P* < 0.001.

| **Source of variation** | **GY** | **TGW** | **BDW** | **SM** | **GS** |
| --- | --- | --- | --- | --- | --- |
| **Y** | 0.003^**^ | 0.004^**^ | 0.000^***^ | 0.012^*^ | 0.000^***^ |
| **N** | 0.045^*^ | 0.229^ns^ | 0.927^ns^ | 0.860^ns^ | 0.798^ns^ |
| **YxN** | 0.121^ns^ | 0.115^ns^ | 0.820^ns^ | 0.709^ns^ | 0.238^ns^ |
| **G** | 0.000^***^ | 0.000^***^ | 0.082^ns^ | 0.000^***^ | 0.000^***^ |
| **GxY** | 0.000^***^ | 0.000^***^ | 0.058^ns^ | 0.258^ns^ | 0.000^***^ |
| **GxN** | 0.617^ns^ | 0.354^ns^ | 0.029* | 0.437^ns^ | 0.346^ns^ |
| **GxYxN** | 0.364^ns^ | 0.166^ns^ | 0.142^ns^ | 0.252^ns^ | 0.482^ns^ |

GY: grain yield (Mg ha^-1^); TGW: Thousand grain weight; BDW: Biomass dry weight; SM: Spikes m^-2^, GS: Grains spike^-1^. * For the 2018/19 season, genotypic means of GY were only calculated for all three fertilizer levels combined because the nitrogen fertilizer treatments had significant effect.

**Supplementary Table 3.** Means ± standard deviation of genotypes for agronomical yield components and biomass. The means followed by different letters are significantly different (p <0.05), according to Tukey's honestly significant difference test (HSD).

| Variety* | 2017/18 | | | | | | | | | | | |
| --- | --- | --- | --- | --- | --- | --- | --- | --- | --- | --- | --- | --- |
|  | GS | | | | | | TGW | | BDW | | SM | |
|  | N50 | | N100 | | N130 | |  |  |  |  |  |  |
| Bologna | 39.0±4.73 | bc | 34.7±0.58 | d | 39.7±7.51 | b | 30.2±2.02 | g | 1.59±0.27 | a | 515±76.54 | a |
| Chambo | 49.0±1.73 | abc | 40.7±6.93 | bcd | 45.3±5.57 | b | 36.7±1.24 | abcd | 1.74±0.27 | a | 472±99.03 | ab |
| Soberbio | 46.7±4.58 | abc | 39.0±1.53 | bcd | 42.3±3.46 | b | 35.2±1.86 | bcd | 1.77±0.36 | a | 489±99.49 | ab |
| Henrik | 41.0±6.56 | bc | 44.7±6.11 | abcd | 42.0±1.53 | b | 37.9±1.05 | ab | 1.84±0.25 | a | 472±67.74 | ab |
| Benchmark | 51.0±4.58 | ab | 51.0±3.79 | ab | 49.0±4.51 | ab | 34.2±1.36 | cde | 1.88±0.34 | a | 461±72.70 | ab |
| RGT Reform | 45.3±4.16 | abc | 42.7±6.24 | abcd | 40.7±7.02 | b | 36.4±1.37 | bcd | 1.84±0.18 | a | 487±44.17 | ab |
| JB Diego | 50.7±4.93 | ab | 47.3±2.52 | abc | 49.0±0.58 | ab | 33.9±2.31 | def | 1.71±0.34 | a | 448±90.20 | ab |
| KWS Lili | 59.0±2.65 | a | 54.0±5.51 | a | 58.7±1.15 | a | 31.2±2.42 | fg | 1.82±0.16 | a | 450±40.38 | ab |
| Hondia | 46.7±5.69 | abc | 43.0±2.00 | abcd | 48.3±2.31 | ab | 39.4±1.55 | a | 1.74±0.33 | a | 375±88.01 | b |
| Julius | 34.0±6.24 | c | 35.3±1.15 | cd | 37.7±2.52 | b | 36.7±2.50 | abc | 1.50±0.23 | a | 428±49.96 | ab |
| CH-Nara | 42.3±7.00 | bc | 42.7±1.73 | abcd | 45.3±4.04 | b | 35.4±1.69 | bcd | 1.60±0.33 | a | 441±67.67 | ab |
| KWS Siskin | 46.0±8.08 | abc | 43.7±3.79 | abcd | 45.3±5.00 | b | 32.2±1.15 | efg | 1.77±0.22 | a | 466±70.80 | ab |
| Variety* | 2018/19 | | | | | | | | | | | |
|  | GS | | | | | | TGW | | BDW | | SM | |
| Bologna | 25.6±4.22 | | | ab | | | 21.06±5.25 | d | 0.64±0.12 | a | 433±58.43 | a |
| Chambo | 26.6±6.11 | | | a | | | 27.57±4.90 | abc | 0.63±0.10 | a | 336±78.00 | abc |
| Soberbio | 23.8±5.14 | | | ab | | | 28.01±2.03 | abc | 0.67±0.16 | a | 348±64.59 | ab |
| Henrik | 23.8±5.85 | | | ab | | | 32.54±2.66 | a | 0.73±0.16 | a | 327±70.38 | bc |
| Benchmark | 21.0±5.59 | | | ab | | | 26.79±3.07 | bc | 0.63±0.13 | a | 257±52.74 | bcd |
| RGT Reform | 19.5±5.75 | | | ab | | | 28.52±2.47 | abc | 0.68±0.17 | a | 317±57.89 | bcd |
| JB Diego | 23.1±5.39 | | | ab | | | 30.06±4.45 | abc | 0.67±0.15 | a | 241±78.67 | cd |
| Bennington | 20.1±8.14 | | | ab | | | 30.63±2.76 | abc | 0.65±0.11 | a | 284±51.69 | bcd |
| Hondia | 16.9±3.81 | | | b | | | 31.95±3.00 | ab | 0.68±0.08 | a | 214±75.88 | d |
| Julius | 19.9±9.02 | | | ab | | | 29.60±2.95 | abc | 0.69±0.17 | a | 285±73.91 | bcd |
| CH-Nara | 21.8±3.26 | | | ab | | | 26.21±3.91 | cd | 0.69±0.11 | a | 306±65.55 | bcd |
| KWS Siskin | 26.5±5.30 | | | a | | | 26.40±2.24 | cd | 0.71±0.21 | a | 307±63.22 | Bcd |

*When the effect of fertilization levels showed a significant difference (p <0.05), the HSD test was performed for each nitrogen level. TGW: Thousand grain weight (g); BDW: Biomass dry weight (kg m^-2^); SM: Spikes m^-2^, GS: Grains spike^-1^.

**Supplementary Table 4.** Summary of the analysis of variance for the year (Y), nitrogen fertilization (N), and genotypic (G) effects and their interactions on phenology (number of days from sowing to reach each of the phenological stages. Levels of significance for the ANOVA: ns, not significant; *, *P* < 0.05; **, *P* < 0.01; ***, *P* < 0.001.

| **Source of variation** | **Tillering** | **Stem elongation** | **Booting** | **Heading** | **Flowering** | **Middle grain filling** | **PEG** |
| --- | --- | --- | --- | --- | --- | --- | --- |
| **Y** | 0.006^**^ | 0.026^*^ | 0.000^***^ | 0.000^***^ | 0.000^***^ | 0.000^***^ | 0.006^ns^ |
| **N** | 0.532^ns^ | 0.721^ns^ | 0.864^ns^ | 0.106^ns^ | 0.188^ns^ | 0.571^ns^ | 0.700^ns^ |
| **YxN** | 0.167^ns^ | 0.864^ns^ | 0.995^ns^ | 0.398^ns^ | 0.689^ns^ | 0.946^ns^ | 0.841^ns^ |
| **G** | 0.054^ns^ | 0.000^***^ | 0.000^***^ | 0.000^***^ | 0.000^***^ | 0.000^***^ | 0.003^**^ |
| **GxY** | 0.034^*^ | 0.000^***^ | 0.000^***^ | 0.000^***^ | 0.000^***^ | 0.000^***^ | 0.000^***^ |
| **GxN** | 0.601^ns^ | 0.535^ns^ | 0.949^ns^ | 0.166^ns^ | 0.254^ns^ | 0.451^ns^ | 0.415^ns^ |
| **GxYxN** | 0.824^ns^ | 0.006^**^ | 0.335^ns^ | 0.268^ns^ | 0.014^*^ | 0.413^ns^ | 0.011^*^ |

PEG: period between the beginning of stem elongation and middle grain filling. Phenological stages were determined visually according to the Zadoks scale (Zadoks et al., 1974).

**Supplementary Table 5.** Means ± standard deviation of genotypes for phenological stages in days after sowing. The means followed by different letters are significantly different (p <0.05), according to Tukey's honestly significant difference test (HSD).

| **Variety** | **2017/2018** | | | | | | | | | | | | | |
| --- | --- | --- | --- | --- | --- | --- | --- | --- | --- | --- | --- | --- | --- | --- |
|  | **Tillering** | | **Stem Elongation** | | **Booting** | | **Heading** | | **Anthesis** | | **Middle grain filling** | | **PEG** | |
| Bologna | 113±4 | a | 134±5 | ab | 189±1 | e | 194±0 | e | 205±3 | e | 223±3 | cd | 88±6 | a |
| Chambo | 112±5 | a | 132±2 | b | 186±1 | f | 192±2 | e | 205±3 | de | 220±1 | d | 88±3 | a |
| Soberbio | 113±5 | a | 135±5 | ab | 189±1 | e | 198±5 | d | 209±4 | bcd | 221±2 | d | 86±6 | a |
| Henrik | 113±1 | a | 139±6 | ab | 200±1 | bc | 206±2 | ab | 214±2 | a | 229±2 | ab | 90±7 | a |
| Benchmark | 115±3 | a | 138±6 | ab | 200±1 | bc | 206±2 | ab | 213±3 | ab | 229±4 | ab | 91±5 | a |
| RGT Reform | 113±4 | a | 135±5 | ab | 201±2 | ab | 207±1 | a | 214±2 | a | 229±4 | ab | 94±7 | a |
| JB Diego | 112±4 | a | 139±6 | ab | 200±1 | bc | 207±2 | ab | 214±3 | a | 230±3 | ab | 90±6 | a |
| KWS Lili | 114±6 | a | 143±7 | a | 200±1 | abc | 208±1 | a | 214±2 | a | 232±1 | a | 89±7 | a |
| Hondia | 114±5 | a | 138±7 | ab | 200±1 | bc | 206±2 | ab | 213±2 | a | 230±5 | ab | 92±6 | a |
| Julius | 113±4 | a | 142±6 | a | 202±2 | a | 208±1 | a | 215±2 | a | 233±2 | a | 91±5 | a |
| CH-Nara | 113±4 | a | 140±7 | ab | 198±1 | d | 202±0 | c | 207±0 | cde | 226±2 | bc | 86±8 | a |
| KWS Siskin | 112±3 | a | 139±7 | ab | 199±1 | cd | 204±1 | bc | 211±2 | abc | 228±3 | ab | 90±7 | a |
|  | **2018/2019** | | | | | | | | | | | | | |
| Bologna | 91±3 | b | 115±3 | e | 155±3 | d | 162±1 | f | 167±1 | c | 191±0 | c | 76±3 | a |
| Chambo | 101±5 | a | 119±5 | de | 155±3 | cd | 163±1 | ef | 169±2 | c | 191±1 | c | 72±5 | abc |
| Soberbio | 97±7 | ab | 119±3 | de | 156±5 | cd | 165±2 | e | 168±2 | c | 191±1 | c | 72±3 | ab |
| Henrik | 95±7 | ab | 127±2 | abc | 165±1 | a | 172±1 | ab | 173±2 | ab | 193±1 | ab | 66±2 | cdef |
| Benchmark | 98±6 | ab | 131±0 | a | 164±2 | a | 172±2 | ab | 173±1 | ab | 193±1 | ab | 62±1 | ef |
| RGT Reform | 99±5 | ab | 133±0 | a | 165±1 | a | 174±2 | a | 174±1 | a | 193±1 | ab | 60±1 | f |
| JB Diego | 100±5 | a | 132±1 | a | 165±1 | a | 171±2 | ab | 174±2 | ab | 194±0 | a | 62±1 | ef |
| Bennington | 99±6 | ab | 128±4 | abc | 164±2 | ab | 170±2 | bc | 173±1 | ab | 193±1 | ab | 65±3 | cdef |
| Hondia | 99±5 | ab | 130±4 | ab | 164±4 | a | 173±3 | ab | 174±2 | a | 193±1 | ab | 63±4 | ef |
| Julius | 96±6 | ab | 130±3 | abc | 165±1 | a | 172±2 | ab | 175±1 | a | 194±1 | ab | 64±3 | def |
| CH-Nara | 98±6 | ab | 125±7 | bcd | 159±3 | bc | 166±1 | de | 172±2 | b | 192±2 | bc | 67±8 | bcde |
| KWS Siskin | 100±6 | a | 124±7 | cd | 163±2 | ab | 168±2 | cd | 173±1 | ab | 193±10 | ab | 69±15 | bcd |

PEG: period between the stem elongation and middle grain filling.

**Supplementary Table 6.** Means ± standard deviation of genotypes for vegetation indices of 2017/18. The means followed by different letters are significantly different (p <0.05), according to Tukey's honestly significant difference test (HSD).

| **Variety*** | **Tillering** | | | | **Stem elongation** | | | | | | | | | | | | | |
| --- | --- | --- | --- | --- | --- | --- | --- | --- | --- | --- | --- | --- | --- | --- | --- | --- | --- | --- |
|  | **GGA1** | | **GGA2** | | **GGA3** | | | | | | **NDVI1** | | **NDVI2** | | | | | |
|  |  |  |  |  | **N50** | | **N100** | | **N130** | |  |  | **N50** | | **N100** | | **N130** | |
| Bologna | 0.026±0.01 | abcd | 0.16±0.05 | abc | 0.41±0.07 | ab | 0.41±0.13 | b | 0.39±0.06 | ab | 0.47±0.05 | abc | 0.58±0.11 | abc | 0.58±0.06 | b | 0.57±0.09 | ab |
| Chambo | 0.036±0.02 | abcd | 0.12±0.05 | abc | 0.34±0.06 | ab | 0.53±0.03 | ab | 0.51±0.11 | ab | 0.47±0.04 | abc | 0.54±0.04 | abc | 0.65±0.03 | ab | 0.64±0.07 | a |
| Soberbio | 0.048±0.03 | a | 0.15±0.12 | abc | 0.53±0.08 | ab | 0.67±0.09 | a | 0.53±0.10 | ab | 0.55±0.10 | ab | 0.70±0.05 | ab | 0.74±0.05 | a | 0.64±0.07 | ab |
| Henrik | 0.028±0.01 | abcd | 0.19±0.04 | ab | 0.45±0.10 | ab | 0.51±0.06 | ab | 0.54±0.14 | a | 0.50±0.06 | abc | 0.65±0.03 | abc | 0.66±0.05 | ab | 0.71±0.02 | a |
| Benchmark | 0.031±0.01 | abcd | 0.21±0.07 | a | 0.45±0.11 | ab | 0.46±0.05 | ab | 0.50±0.11 | ab | 0.48±0.08 | abc | 0.63±0.08 | abc | 0.64±0.02 | ab | 0.66±0.13 | a |
| RGT Reform | 0.024±0.01 | bcd | 0.21±0.04 | a | 0.43±0.02 | ab | 0.58±0.03 | ab | 0.52±0.06 | ab | 0.49±0.09 | abc | 0.64±0.02 | abc | 0.71±0.00 | ab | 0.64±0.04 | a |
| JB Diego | 0.040±0.02 | abc | 0.07±0.07 | c | 0.62±0.13 | a | 0.69±0.01 | a | 0.61±0.04 | a | 0.57±0.08 | a | 0.75±0.09 | a | 0.78±0.05 | a | 0.71±0.04 | a |
| KWS Lili | 0.017±0.01 | d | 0.08±0.03 | c | 0.26±0.06 | b | 0.51±0.07 | ab | 0.48±0.06 | ab | 0.43±0.06 | c | 0.53±0.04 | bc | 0.69±0.01 | ab | 0.65±0.05 | a |
| Hondia | 0.033±0.01 | abcd | 0.11±0.04 | bc | 0.44±0.22 | ab | 0.57±0.13 | ab | 0.56±0.05 | a | 0.50±0.03 | abc | 0.66±0.13 | abc | 0.72±0.11 | ab | 0.67±0.01 | a |
| Julius | 0.019±0.01 | cd | 0.10±0.04 | bc | 0.37±0.07 | ab | 0.50±0.08 | ab | 0.55±0.12 | a | 0.45±0.05 | bc | 0.57±0.07 | abc | 0.67±0.06 | ab | 0.69±0.04 | a |
| CH-Nara | 0.023±0.01 | bcd | 0.12±0.03 | abc | 0.30±0.08 | b | 0.42±0.13 | b | 0.28±0.06 | b | 0.40±0.06 | c | 0.47±0.04 | c | 0.57±0.06 | b | 0.46±0.06 | b |
| KWS Siskin | 0.045±0.02 | ab | 0.16±0.09 | abc | 0.49±0.04 | ab | 0.68±0.03 | a | 0.57±0.08 | a | 0.55±0.04 | a | 0.69±0.09 | ab | 0.74±0.04 | a | 0.70±0.02 | a |
|  | **Booting** | | | | | | | | | | | | **Anthesis** | | | | | |
|  | **GGA4** | | | | | | **NDVI3** | | | | | | **GGA5** | | | | | |
|  | **N50** | | **N100** | | **N130** | | **N50** | | **N100** | | **N130** | | **N50** | | **N100** | | **N130** | |
| Bologna | 0.72±0.04 | ab | 0.70±0.03 | bc | 0.65±0.05 | ab | 0.69±0.00 | a | 0.69±0.05 | ab | 0.68±0.04 | a | 0.71±0.02 | abc | 0.69±0.03 | c | 0.70±0.07 | bc |
| Chambo | 0.59±0.04 | ab | 0.72±0.05 | abc | 0.77±0.08 | ab | 0.54±0.06 | ab | 0.65±0.02 | ab | 0.70±0.07 | a | 0.57±0.05 | c | 0.74±0.04 | bc | 0.79±0.05 | ab |
| Soberbio | 0.69±0.07 | ab | 0.77±0.03 | abc | 0.71±0.11 | ab | 0.63±0.02 | a | 0.71±0.07 | a | 0.66±0.06 | a | 0.74±0.09 | abc | 0.88±0.04 | a | 0.82±0.04 | ab |
| Henrik | 0.65±0.04 | ab | 0.76±0.06 | abc | 0.75±0.11 | ab | 0.52±0.03 | ab | 0.62±0.07 | ab | 0.62±0.08 | ab | 0.76±0.06 | ab | 0.85±0.05 | ab | 0.84±0.04 | a |
| Benchmark | 0.66±0.09 | ab | 0.65±0.07 | c | 0.76±0.07 | ab | 0.53±0.10 | ab | 0.55±0.07 | b | 0.61±0.06 | ab | 0.81±0.09 | ab | 0.76±0.05 | abc | 0.82±0.05 | ab |
| RGT Reform | 0.67±0.09 | ab | 0.76±0.02 | abc | 0.66±0.01 | ab | 0.55±0.04 | ab | 0.63±0.04 | ab | 0.59±0.04 | ab | 0.78±0.08 | ab | 0.86±0.03 | a | 0.86±0.06 | a |
| JB Diego | 0.79±0.05 | a | 0.85±0.06 | a | 0.74±0.06 | ab | 0.66±0.03 | a | 0.71±0.03 | ab | 0.64±0.04 | ab | 0.84±0.02 | a | 0.86±0.05 | a | 0.82±0.05 | ab |
| KWS Lili | 0.56±0.02 | b | 0.76±0.06 | abc | 0.81±0.05 | a | 0.54±0.06 | ab | 0.67±0.05 | ab | 0.71±0.03 | a | 0.67±0.04 | abc | 0.84±0.03 | ab | 0.84±0.04 | a |
| Hondia | 0.68±0.07 | ab | 0.78±0.06 | abc | 0.81±0.06 | a | 0.59±0.11 | ab | 0.68±0.07 | ab | 0.66±0.04 | a | 0.78±0.11 | ab | 0.86±0.03 | a | 0.85±0.04 | a |
| Julius | 0.69±0.13 | ab | 0.79±0.08 | abc | 0.82±0.01 | a | 0.60±0.10 | ab | 0.70±0.03 | ab | 0.68±0.05 | a | 0.78±0.04 | ab | 0.87±0.07 | a | 0.88±0.03 | a |
| CH-Nara | 0.61±0.06 | ab | 0.70±0.04 | bc | 0.60±0.06 | b | 0.43±0.03 | b | 0.56±0.07 | ab | 0.50±0.09 | b | 0.64±0.02 | bc | 0.70±0.02 | c | 0.66±0.03 | c |
| KWS Siskin | 0.69±0.08 | ab | 0.81±0.03 | ab | 0.82±0.05 | a | 0.56±0.05 | ab | 0.63±0.04 | ab | 0.67±0.01 | a | 0.80±0.02 | ab | 0.87±0.01 | a | 0.87±0.00 | a |
|  | **Anthesis** | | | | | | **Middle grain filling** | | | | | | | | | | | |
|  | **NDVI4** | | | | | | **GGA6** | | | | | | **NDVI5** | | | | | |
|  | **N50** | | **N100** | | **N130** | | **N50** | | **N100** | | **N130** | | **N50** | | **N100** | | **N130** | |
| Bologna | 0.74±0.02 | ab | 0.74±0.02 | ab | 0.73±0.02 | ab | 0.27±0.08 | cd | 0.28±0.03 | f | 0.26±0.03 | c | 0.60±0.03 | ab | 0.58±0.03 | cd | 0.62±0.04 | ab |
| Chambo | 0.62±0.06 | ab | 0.72±0.01 | abc | 0.73±0.04 | ab | 0.23±0.12 | d | 0.29±0.06 | f | 0.38±0.09 | c | 0.48±0.06 | bc | 0.53±0.02 | d | 0.59±0.06 | ab |
| Soberbio | 0.71±0.01 | ab | 0.76±0.02 | a | 0.74±0.03 | a | 0.28±0.08 | cd | 0.38±0.05 | ef | 0.41±0.09 | c | 0.59±0.02 | ab | 0.64±0.04 | abc | 0.62±0.02 | ab |
| Henrik | 0.60±0.02 | b | 0.65±0.03 | d | 0.65±0.03 | bc | 0.54±0.02 | ab | 0.71±0.07 | ab | 0.62±0.10 | ab | 0.52±0.04 | abc | 0.60±0.03 | bcd | 0.60±0.04 | ab |
| Benchmark | 0.66±0.05 | ab | 0.69±0.03 | bcd | 0.70±0.03 | abc | 0.57±0.07 | ab | 0.58±0.04 | cd | 0.63±0.02 | a | 0.60±0.08 | ab | 0.62±0.03 | abcd | 0.64±0.05 | ab |
| RGT Reform | 0.70±0.03 | ab | 0.74±0.01 | ab | 0.72±0.04 | ab | 0.60±0.08 | ab | 0.69±0.03 | abc | 0.68±0.07 | a | 0.63±0.03 | a | 0.71±0.01 | a | 0.68±0.05 | ab |
| JB Diego | 0.72±0.03 | ab | 0.75±0.03 | ab | 0.73±0.01 | ab | 0.59±0.07 | ab | 0.65±0.08 | abc | 0.63±0.07 | ab | 0.65±0.03 | a | 0.68±0.05 | abc | 0.66±0.03 | ab |
| KWS Lili | 0.62±0.14 | ab | 0.71±0.01 | abc | 0.72±0.01 | ab | 0.48±0.11 | abc | 0.64±0.01 | bc | 0.67±0.04 | a | 0.54±0.05 | abc | 0.65±0.04 | abc | 0.68±0.04 | a |
| Hondia | 0.77±0.04 | a | 0.75±0.03 | ab | 0.73±0.02 | ab | 0.56±0.05 | ab | 0.66±0.01 | abc | 0.66±0.04 | a | 0.62±0.05 | a | 0.69±0.05 | abc | 0.67±0.03 | ab |
| Julius | 0.66±0.07 | ab | 0.74±0.01 | ab | 0.74±0.05 | a | 0.63±0.10 | a | 0.79±0.06 | a | 0.74±0.04 | a | 0.60±0.07 | ab | 0.68±0.03 | abc | 0.67±0.04 | ab |
| CH-Nara | 0.59±0.02 | b | 0.66±0.03 | cd | 0.62±0.04 | c | 0.38±0.06 | bcd | 0.47±0.02 | de | 0.44±0.10 | bc | 0.45±0.02 | c | 0.54±0.05 | d | 0.54±0.10 | b |
| KWS Siskin | 0.70±0.04 | ab | 0.74±0.02 | ab | 0.75±0.03 | a | 0.56±0.03 | ab | 0.68±0.01 | abc | 0.66±0.02 | a | 0.62±0.05 | a | 0.70±0.05 | ab | 0.69±0.03 | a |

*When the effect of fertilization levels showed a significant difference (p <0.05), the HSD test was performed for each nitrogen level. TGW: Thousand grain weight (g); BDW: Biomass dry weight (kg m^-2^); SM: Spikes m^-2^, GS: Grains spike^-1^.

**Supplementary Table 7.** Means ± standard deviation of genotypes for vegetation indices of 2018/19. The means followed by different letters are significantly different (p <0.05), according to Tukey's honestly significant difference test (HSD).

| **Variety** | **3 leaves** | | **Tillering** | | | | | | **Stem elongation** | | | | | | | |
| --- | --- | --- | --- | --- | --- | --- | --- | --- | --- | --- | --- | --- | --- | --- | --- | --- |
|  | **GGA1** | | **GGA2** | | **GGA3** | | **NDVI1** | | **GGA4** | | **GGA5** | | **NDVI2** | | **NDVI3** | |
| Bologna | 0.011±0.00 | b | 0.17±0.04 | b | 0.42±0.11 | a | 0.50±0.11 | ab | 0.53±0.08 | a | 0.65±0.06 | bc | 0.56±0.08 | ab | 0.63±0.06 | cd |
| Chambo | 0.012±0.01 | b | 0.14±0.06 | b | 0.39±0.11 | a | 0.42±0.09 | b | 0.54±0.09 | a | 0.63±0.04 | c | 0.53±0.08 | b | 0.61±0.06 | d |
| Soberbio | 0.020±0.00 | a | 0.29±0.06 | a | 0.54±0.09 | a | 0.60±0.09 | a | 0.68±0.09 | a | 0.75±0.07 | ab | 0.65±0.05 | a | 0.70±0.05 | abc |
| Henrik | 0.015±0.00 | ab | 0.17±0.06 | b | 0.45±0.11 | a | 0.52±0.10 | ab | 0.63±0.11 | a | 0.76±0.07 | a | 0.63±0.09 | ab | 0.72±0.05 | ab |
| Benchmark | 0.02±0.010 | a | 0.20±0.10 | ab | 0.50±0.12 | a | 0.54±0.10 | ab | 0.66±0.11 | a | 0.75±0.07 | ab | 0.62±0.08 | ab | 0.68±0.05 | abcd |
| RGT Reform | 0.012±0.00 | b | 0.14±0.05 | b | 0.36±0.10 | a | 0.45±0.10 | b | 0.59±0.11 | a | 0.71±0.06 | abc | 0.58±0.06 | ab | 0.69±0.04 | abcd |
| JB Diego | 0.013±0.00 | b | 0.18±0.07 | b | 0.49±0.08 | a | 0.55±0.07 | ab | 0.64±0.06 | a | 0.77±0.06 | a | 0.63±0.06 | ab | 0.72±0.05 | ab |
| Bennington | 0.014±0.00 | ab | 0.21±0.07 | ab | 0.48±0.13 | a | 0.57±0.10 | ab | 0.65±0.09 | a | 0.79±0.08 | a | 0.62±0.08 | ab | 0.69±0.05 | abc |
| Hondia | 0.015±0.00 | ab | 0.19±0.06 | b | 0.47±0.12 | a | 0.54±0.08 | ab | 0.65±0.12 | a | 0.77±0.07 | a | 0.65±0.04 | a | 0.73±0.02 | a |
| Julius | 0.017±0.00 | ab | 0.17±0.09 | b | 0.44±0.11 | a | 0.48±0.08 | ab | 0.63±0.10 | a | 0.79±0.07 | a | 0.61±0.07 | ab | 0.73±0.04 | a |
| CH-Nara | 0.016±0.00 | ab | 0.20±0.06 | ab | 0.44±0.12 | a | 0.49±0.08 | ab | 0.59±0.12 | a | 0.72±0.08 | abc | 0.56±0.08 | ab | 0.65±0.05 | bcd |
| KWS Siskin | 0.015±0.00 | ab | 0.19±0.08 | ab | 0.49±0.11 | a | 0.52±0.09 | ab | 0.66±0.09 | a | 0.77±0.07 | a | 0.63±0.07 | ab | 0.72±0.06 | ab |
|  | **Booting** | | | | **Heading** | | | | **Anthesis** | | | | | | **Middle grain filling** | |
|  | **GGA6** | | **NDVI4** | | **GGA7** | | **NDVI5** | | **GGA8** | | **GGA9** | | **NDVI6** | | **GGA10** | |
| Bologna | 0.53±0.08 | b | 0.45±0.07 | d | 0.32±0.07 | c | 0.44±0.06 | abc | 0.06±0.05 | e | 0.06±0.31 | d | 0.33±0.04 | ab | 0.33±0.30 | a |
| Chambo | 0.53±0.13 | ab | 0.46±0.08 | cd | 0.33±0.11 | bc | 0.40±0.08 | c | 0.10±0.07 | de | 0.07±0.13 | d | 0.30±0.05 | ab | 0.36±0.14 | a |
| Soberbio | 0.68±0.09 | a | 0.51±0.04 | abcd | 0.36±0.10 | bc | 0.45±0.05 | abc | 0.13±0.05 | cde | 0.18±0.09 | cd | 0.33±0.04 | ab | 0.28±0.04 | a |
| Henrik | 0.63±0.08 | ab | 0.54±0.05 | abcd | 0.49±0.08 | ab | 0.40±0.06 | c | 0.30±0.06 | a | 0.67±0.08 | a | 0.33±0.04 | ab | 0.33±0.13 | a |
| Benchmark | 0.66±0.10 | ab | 0.49±0.05 | bcd | 0.39±0.09 | abc | 0.53±0.05 | a | 0.19±0.08 | bcd | 0.33±0.14 | bcd | 0.29±0.04 | b | 0.13±0.32 | a |
| RGT Reform | 0.59±0.12 | ab | 0.52±0.07 | abcd | 0.38±0.12 | abc | 0.41±0.07 | bc | 0.23±0.10 | abc | 0.29±0.21 | bcd | 0.31±0.05 | ab | 0.28±0.25 | a |
| JB Diego | 0.64±0.11 | ab | 0.55±0.07 | abcd | 0.44±0.12 | abc | 0.46±0.07 | abc | 0.21±0.11 | abcd | 0.28±0.25 | bcd | 0.33±0.06 | ab | 0.26±0.42 | a |
| Bennington | 0.65±0.10 | ab | 0.51±0.07 | abcd | 0.38±0.10 | abc | 0.39±0.07 | c | 0.17±0.07 | bcd | 0.18±0.28 | cd | 0.29±0.05 | b | 0.21±0.34 | a |
| Hondia | 0.67±0.11 | ab | 0.58±0.06 | ab | 0.49±0.10 | ab | 0.41±0.07 | bc | 0.27±0.09 | ab | 0.57±0.32 | ab | 0.37±0.07 | a | 0.02±0.36 | a |
| Julius | 0.62±0.08 | ab | 0.60±0.08 | a | 0.53±0.09 | a | 0.50±0.07 | ab | 0.26±0.06 | ab | 0.47±0.14 | abc | 0.37±0.05 | a | 0.29±0.33 | a |
| CH-Nara | 0.58±0.09 | ab | 0.48±0.05 | cd | 0.39±0.11 | abc | 0.43±0.05 | bc | 0.21±0.07 | abcd | 0.26±0.19 | bcd | 0.29±0.04 | b | 0.17±0.31 | a |
| KWS Siskin | 0.67±0.12 | ab | 0.55±0.07 | abc | 0.41±0.09 | abc | 0.44±0.07 | abc | 0.22±0.05 | abc | 0.32±0.21 | bcd | 0.34±0.03 | ab | 0.08±0.23 | a |

**Supplementary Table 8.** Pearson's correlation coefficient of grain yield with yield components, phenological stages, and the vegetation indices greener area (GGA), and normalized difference vegetation index (NDVI) (n=108).

| **Traits** | **2017/18** | | **2018/19** | |
| --- | --- | --- | --- | --- |
| TGW | 0.03 | ^ns^ | -0.16 | ^ns^ |
| BDW | -0.01 | ^ns^ | 0.01 | ^ns^ |
| SM | 0.12 | ^ns^ | 0.57 | ^**^ |
| GS | 0.12 | ^ns^ | 0.53 | ^**^ |
| NDVI1 | 0.61 | ^**^ | -0.10 | ^ns^ |
| NDVI2 | 0.72 | ^**^ | 0.02 | ^ns^ |
| NDVI3 | 0.45 | ^**^ | 0.06 | ^ns^ |
| NDVI4 | 0.44 | ^**^ | 0.18 | ^ns^ |
| NDVI5 | 0.66 | ^**^ | 0.29 | ^**^ |
| NDVI6 |  |  | 0.26 | ^**^ |
| GGA1 | 0.41 | ^**^ | -0.03 | ^ns^ |
| GGA2 | 0.46 | ^**^ | 0.10 | ^ns^ |
| GGA3 | 0.72 | ^**^ | 0.05 | ^ns^ |
| GGA4 | 0.56 | ^**^ | -0.04 | ^ns^ |
| GGA5 | 0.67 | ^**^ | -0.07 | ^ns^ |
| GGA6 | 0.53 | ^**^ | -0.07 | ^ns^ |
| GGA7 |  |  | -0.04 | ^ns^ |
| GGA8 |  |  | -0.06 | ^ns^ |
| GGA9 |  |  | 0.22 | ^*^ |
| GGA10 |  |  | 0.19 | ^*^ |
| Tillering | -0.27 | ^**^ | 0.06 | ^ns^ |
| Stem elongation | -0.12 | ^ns^ | -0.34 | ^**^ |
| Booting | 0.18 | ^ns^ | -0.34 | ^**^ |
| Heading | 0.28 | ^**^ | -0.40 | ^**^ |
| Anthesis | 0.36 | ^**^ | -0.34 | ^**^ |
| Middle grain filling | 0.14 | ^ns^ | -0.22 | ^*^ |

**
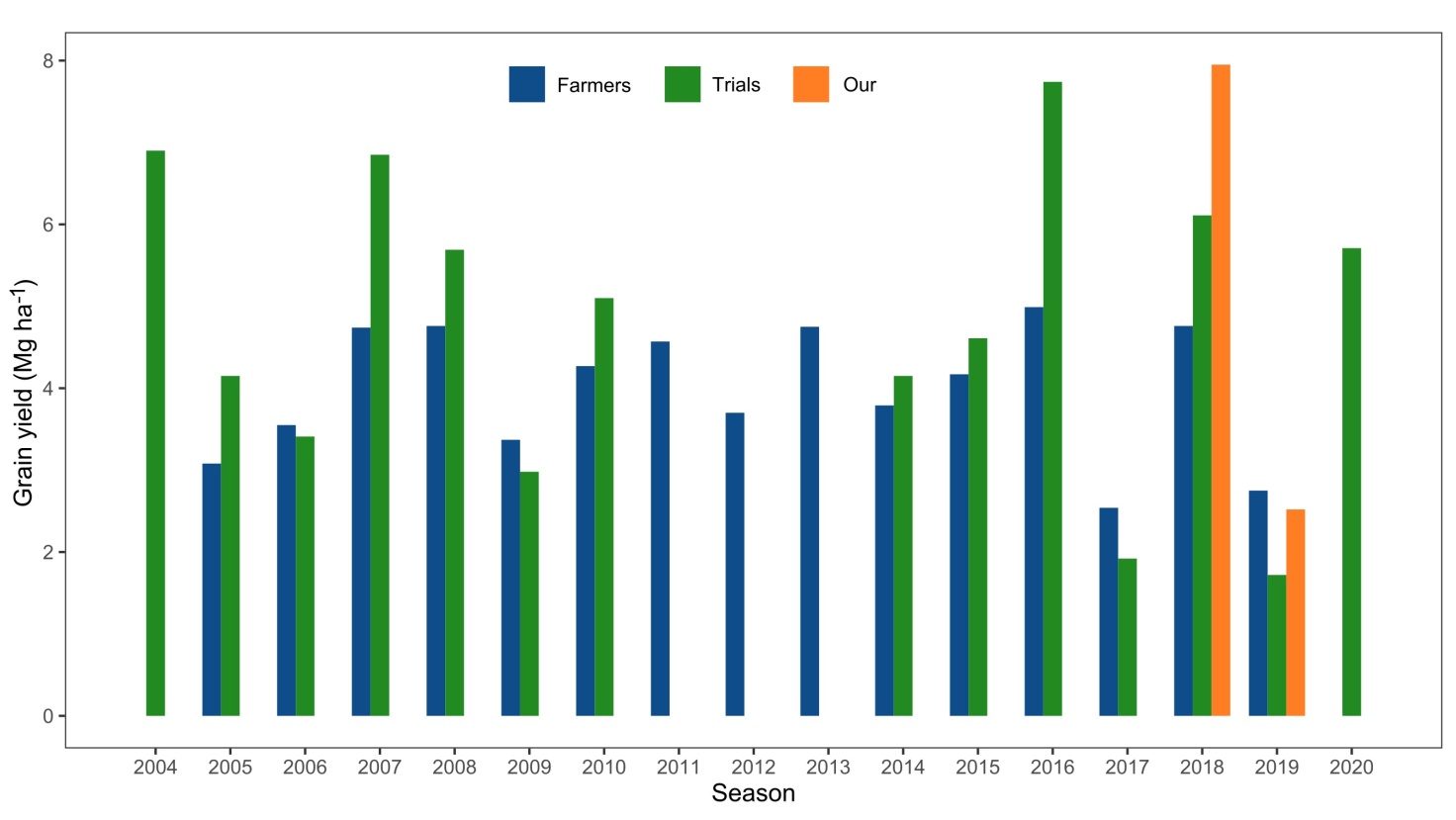
**

**Supplementary Figure 1.** Comparison of the average grain yield of two years of evaluation of our experiment (Our) with the yield of commercial cultivars of the farmers (Farmers) and experimental trials of new registrated varieties (Trials) carry out by ITACyL (Instituto Tecnológico Agrario de Castilla y León) across different environments, between 2004 and 2020, for the region of Castilla y León, Spain.
